# Computational Insight Into Vitamin K_1_ ω-Hydroxylation by Cytochrome P450 4F2

**DOI:** 10.3389/fphar.2018.01065

**Published:** 2018-09-25

**Authors:** Junhao Li, Hongxiao Zhang, Guixia Liu, Yun Tang, Yaoquan Tu, Weihua Li

**Affiliations:** ^1^Shanghai Key Laboratory of New Drug Design, School of Pharmacy, East China University of Science and Technology, Shanghai, China; ^2^Department of Theoretical Chemistry and Biology, School of Engineering Sciences in Chemistry, Biotechnology and Health (CBH), KTH Royal Institute of Technology, Stockholm, Sweden

**Keywords:** cytochrome P450, CYP4F2, ω-hydroxylation, molecular dynamics, QM/MM, homology modeling, vitamin K_1_

## Abstract

Vitamin K_1_ (VK1) plays an important role in the modulation of bleeding disorders. It has been reported that ω-hydroxylation on the VK1 aliphatic chain is catalyzed by cytochrome P450 4F2 (CYP4F2), an enzyme responsible for the metabolism of eicosanoids. However, the mechanism of VK1 ω-hydroxylation by CYP4F2 has not been disclosed. In this study, we employed a combination of quantum mechanism (QM) calculations, homology modeling, molecular docking, molecular dynamics (MD) simulations, and combined quantum mechanism/molecular mechanism (QM/MM) calculations to investigate the metabolism profile of VK1 ω-hydroxylation. QM calculations based on the truncated VK1 model show that the energy barrier for ω-hydroxylation is about 6-25 kJ/mol higher than those at other potential sites of metabolism. However, results from the MD simulations indicate that hydroxylation at the ω-site is more favorable than at the other potential sites, which is in accordance with the experimental observation. The evaluation of MD simulations was further endorsed by the QM/MM calculation results. Our studies thus suggest that the active site residues of CYP4F2 play a determinant role in the ω-hydroxylation. Our results provide structural insights into the mechanism of VK1 ω-hydroxylation by CYP4F2 at the atomistic level and are helpful not only for characterizing the CYP4F2 functions but also for looking into the ω-hydroxylation mediated by other CYP4 enzymes.

## Introduction

The cytochrome P450 enzyme family 4 (CYP4) has 12 members and is the second largest human CYP family. Most of the CYP4 enzymes are ω-hydroxylases except for CYP4F8, 4F12, 4X1, and 4Z1 (Hardwick, 2008; Edson and Rettie, 2013). A wide range of compounds with saturated or unsaturated chains, such as fatty acids, eicosanoids, dietary phytanic acid, drugs, and vitamins E and K (Hsu et al., 2007; Hardwick, 2008; Edson and Rettie, 2013), have been found to be substrates of CYP4 ω-hydroxylases. Some of the substrates are physiologically important eicosanoids and related to the Refsum disease and X-linked adrenoleukodystrophy (X-ALD) (Hardwick, 2008; Edson and Rettie, 2013).

In ω-hydroxylation, an oxygen is inserted into the terminal C–H bond on an inactive aliphatic chain (Björkhem, 1978). This is unusual to some CYP2 enzymes that hydroxylate the internal sites(ω-1, ω-2 sites, etc.) on the chain, where the C–H bonds at the ω site are chemically stronger than those at the internal sites (Shilov and Shul’pin, 1997; Olsen et al., 2006). However, it was found that CYP4 ω-hydroxylation does not necessarily occur at the ω site. It can take place at some internal sites. The hydroxylation ratio between the ω and the internal sites spans from more than 20:1 to 0:1 and varies significantly for different CYP4 enzyme/substrate pairs (Hsu et al., 2017). The hydroxylation occurrence at the internal sites might be ascribed to the shapes and volumes of different CYP4 hydroxylase, which enables these enzymes to catalyze the ω-hydroxylation of short chain (C7-C10), medium chain (C10-C16), long chain (C17-C21), and very long chain (C22-C26) substrates. For example, CYP4B1 only specifically ω-hydroxylates short chain saturate alkanes (Fisher et al., 1998; Hsu et al., 2017).

The most import structural feature for the uncommon ω-hydroxylation is assumed to be the unique covalent link between the 5-methyl group of heme and the carboxyl group of a glutamic acid in helix I, which was indirectly confirmed by UV spectra studies (Hoch et al., 2000; Hoch et al., 2001; Baer et al., 2005; Ortiz and de Montellano, 2008). The ester bond between the heme and glutamic acid could not be identified in the UV spectra when the glutamic acid was mutated to alanine (LeBrun et al., 2002). Nevertheless, the crystal structure of the P450 BM3 (CYP102A1) A264E mutant, which is located in the similar flanking position of helix I, reveals that the carboxyl group of Glu264 ligates to the iron rather than covalently bonds to the 5-methyl group of the heme as expected (Joyce et al., 2004). These studies suggested that the ester bond between a specific glutamic acid in helix I and heme is formed inherently and automatically in the CYP4 enzymes (LeBrun et al., 2002). This assumption was further confirmed by the crystal structure of rabbit CYP4B1 (Hsu et al., 2017), which shows that there exists an ester bond between the Glu310 and heme directly. The E310D and E310Q mutants exhibit reduced hydroxylation rates at the ω, ω-1, and ω-2 sites. However, the ratio of ω/ω-1 hydroxylation increases significantly in these mutants, indicating that although the covalent bond between Glu310 and heme could reduce the conformational entropies in the active site (Hsu et al., 2017), the existence of this covalent bond is not necessary in the selective ω-hydroxylation of short chain alkanes.

To understand the role of active site residues and substrate reactivity in the ω-hydroxylation mediated by CYP4 enzymes, vitamin K1 (VK1) ω-hydroxylation mediated by CYP4F2 was investigated in detail in this work. CYP4F2 is responsible for the metabolism of long chain fatty acids with unsaturated and/or branched chains, such as arachidonic acid, leukotriene-B4, and menaquinone-4 (Kikuta et al., 2000). It has been reported that its genetic polymorphism (V433M) is associated with the increased dose response to warfarin through the ω-hydroxylation of several forms of vitamin K, which are important in the synthesis of coagulator (McDonald et al., 2009; Edson et al., 2013). In this study, quantum mechanism (QM) calculations were first performed to compare the C–H activation energies of different sites with two conformers using a truncated VK1 model. A homology model of human CYP4F2 was generated using the crystal structure of rabbit CYP4B1 as the template. Molecular docking and molecular dynamics (MD) simulations were then carried out to investigate the effect of the active site residues of CYP4F2 on the hydroxylation of VK1. Based on the evaluation of MD simulations, combined quantum mechanism/molecular mechanism (QM/MM) calculations were carried out to justify the selectivity of VK1 hydroxylation by CYP4F2.

## Materials and Methods

### QM Calculations

**FIGURE 1:**
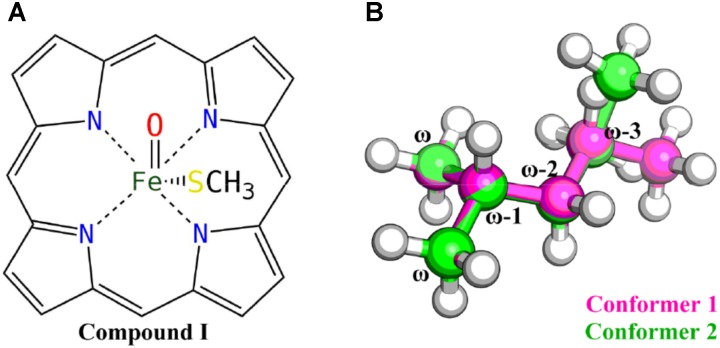
Truncated Cpd I **(A)** and VK1 **(B)** models used in the QM calculations.

All QM calculations were carried out at the density functional theory (DFT) level using the Gaussian 09 package (G09 D.01) (Frisch et al., 2013). The B3LYP functional (Lee et al., 1988; Becke, 1993), which has been extensively used in the studies of heme-containing enzyme systems (Shaik et al., 2010; Rydberg et al., 2014), was chosen for the geometry optimizations. The 6-31G(d) basis set was used for all the atoms except the iron, for which the relativistic LANL2DZ pseudopotential and associated basis set was used (Hay and Wadt, 1985). The single point energy was computed using the dispersion corrected B3LYP-D3 functional (Grimme et al., 2010) with Becke–Johnson damping (Grimme et al., 2011), using the LANL2DZ pseudopotential and associated basis set for iron and the 6-311 + G(d,p) basis set for other atoms. The solvation effect was implicitly considered using the polarizable continuum model (Cancès et al., 1997) in the final energy calculations with the dielectric constant assigned to 4. The zero-point energy (ZPE) corrections were estimated from vibrational frequencies calculations using the same level of theory and basis sets as employed in geometry optimizations with the temperature set to 298.15 K. The heme moiety was modeled as the compound I (Cpd I) (Shaik et al., 2010) species without side chains, and the cysteine ligated to the iron was modeled by a methyl mercaptide group (**Figure 1A**). Mass-weighted intrinsic reaction coordinate (IRC) calculations (Gonzalez and Schlegel, 1990) were carried out to ensure that the transition structure indeed connected the appropriate reactant and product and to locate the geometry of the reactant complex. Since there are a bunch of VK1 conformers due to its flexible aliphatic chain, only the last six carbon atoms of the aliphatic chain were kept, resulting in an isohexane molecule with only two conformers considered (**Figure 1B**).

### Homology Modeling of CYP4F2

The crystal structure of rabbit CYP4B1 (PDB code: 5T6Q) (Hsu et al., 2017; Scott, 2017), which is the only structure of mammalian CYP4 available at present, was used to build the CYP4F2 model. The sequences of rabbit CYP4B1 (CYP4B1, Uniprot ID: P15128) and human CYP4F2 (CYP4F2, Uniprot ID: P78329) was aligned in ClustalX 2.0 (Thompson et al., 1994). The template structure was pretreated by the prepare-wizard module of Schrödinger (Schrödinger, LLC: New York, NY, 2016). The missing coordinates of residues spanning from Asp196 to Asn200 and Ile272 to Arg276 were automatically rebuilt using the IMAPCT module of Schrödinger (Impact, S. LLC, New York, NY, 2016). Modeller 9v9 (Šali et al., 1995) was used to generate 500 models of CYP4F2 based on the sequence alignment. These models were then evaluated by the DOPE (Discrete Optimized Protein Energy) assessment method (Shen and Sali, 2006) implemented in Modeller. The best-scored model was further validated by the VERIFY_3D (Bowie et al., 1991) and ERRAT (Colovos and Yeates, 1993) modules implemented in the Structural Analysis and Verification Server (SAVES 5.0) ^1^.

### Model Refinement by MD Simulation

The initial CYP4F2 model was refined by MD simulation using Gromacs 5.0.4 (Berendsen et al., 1995; Spoel et al., 2005). The CHARMM36 force field (Huang and MacKerell, 2013) was applied. The force field parameters for the covalent bond between Glu328 and heme (the OE–CMD bond) were adopted from the general CHARMM force field (version 3) (Vanommeslaeghe and MacKerell, 2012; Vanommeslaeghe et al., 2012) with slight modifications of the original heme parameters (**Supplementary Tables S1, S2** and **Supplementary Figure S1**). The heme oxo-iron (IV) complex (Cpd I) was used to mimic the final step in the P450 catalytic cycle as reported in recent studies (Dodani et al., 2016; Bonomo et al., 2017a,b; Onoda et al., 2018; Zheng et al., 2018). The protonation states of the ionizable residues were determined by PROPKA 2.0 (Li et al., 2005). According to the prediction of PROPKA, His118, His127, His348, and His466 were protonated on both the δ and the 𝜀 positions, His34, His74, His165, His235, His363, and His372 were protonated on the 𝜀 position, and other histidine residues were protonated on the δ position. The structure thus obtained was solvated in a dodecahedron box of TIP3P water molecules extending 10 Å from any atoms of the protein in any directions. Finally, two sodium ions were added to keep the system neutral.

The steepest descent method was carried out to minimize the system in the first three steps corresponding to harmonic restraints on the non-water atoms, the protein heavy atoms, and the main chain atoms of the protein, respectively. The final minimization step was accomplished without any restraint. The system was then heated to 300 K using the *v-rescale* temperature coupling scheme (Bussi et al., 2007) using the NVT ensemble in 100 ps, followed by another 100-ps NPT simulation using the Parrinello–Rahman pressure coupling scheme (Parrinello and Rahman, 1981). Finally, a 100-ns unrestrained production simulation using the NPT ensemble was conducted. The LINCS algorithm was adopted to constrain all bonds involving hydrogen atoms during the MD simulations (Hess et al., 1997). The cutoff for the non-bonded interactions was 12 Å. The Particle Mesh Ewald method (Essmann et al., 1995) was applied to recover the long-range electrostatic interaction with the grid spacing assigned to 1.0 Å. A time step of 2 fs was used and the trajectory was saved every 2 ps. For the ligand-free CYP4F2 model, three independent 100-ns MD simulations were performed using random initial velocities. The MD trajectories were clustered using the gromos method (Daura et al., 1999) implemented in Gromacs.

### Molecular Docking

The representative snapshot from the largest cluster of each MD simulation was energy minimized with Gromacs as described above. The initial structure of VK1 was downloaded from Drugbank 4.0 (Law et al., 2014) and prepared using the LigPrep module of Schrödinger. Docking of VK1 to the three energy-minimized structures with the Cpd I was accomplished with GOLD 5.2.2 (Jones et al., 1997) using a procedure similar to that in our previous study (Li et al., 2016). The structures were superimposed to the crystal structure of CYP4B1, and the centroid coordinate of the ligand in CYP4B1 was defined as the grid center for docking. Residues within 25 Å from the grid center were considered as the binding pocket. The output poses were clustered at a threshold of 1.5 Å.

### MD Simulation of the VK1-CYP4F2 Complex

Three independent 400-ns MD simulations were carried out for the selected docked VK1-CYP4F2 complex using the same protocol as described earlier. The force field parameters of VK1 were derived from the CGenFF force field (Vanommeslaeghe et al., 2010). The charge penalties for the aliphatic chain of VK1 were small (ranging from 0 to 3 for the aliphatic chain atoms), since there exist similar templates in the CHARMM force field. The results of the MD simulations were analyzed using VMD 1.9.3 (Humphrey et al., 1996).

### QM/MM Simulation

To justify the results of the truncated QM model and MD simulations, we further performed QM calculations using the ONIOM model (Dapprich et al., 1999) on an MD snapshot involving the RC between ω and ω-1 sites. The ONIOM calculations were performed using Gaussian09 (Rev. D01). The TAO package (Tao and Schlegel, 2010) was used to construct the ONIOM model. The representative snapshot was first energy-minimized using the *pmemd* module of Amber 16 (Case et al., 2016), followed by the ONIOM geometry optimization. Flexible scanning on the potential energy surface was applied to locate the transition states of the ω and ω-1 sites. The protein, VK1, and water and ions within 5 Å of the protein were kept for the ONIOM calculations, resulting in a system with about 16,000 atoms. Residues and water molecules within 8 Å of VK1 were allowed to move freely, while the other atoms were frozen during the optimization. The side chain atoms of Leu137, Phe327, Glu328, and Thr332, the truncated Cpd I, and the isohexane tail of VK1, were included in the QM region. The multiplicity of the system was set to 4. Atoms in the QM regions were treated with the same level of theory by using the same basis sets for both geometry optimization and single point energy calculation as described in the QM calculations, except that the polarizable continuum model was excluded due to the explicit solvent environment in QM/MM calculation (Chung et al., 2015). The AMBER force field was employed for molecular mechanics calculations (See **Supplementary File** for the detail). The electronic embedding scheme was not considered in the single point energy calculations (Li et al., 2017).

## Results and Discussion

### C–H Hydroxylation Selectivity Prediction Based on QM Calculations

The hydrogen atom transfer (HAT) is generally the first and rate-limiting step for the aliphatic hydroxylation, which is one of the most common types of metabolism mediated by CYP enzymes (Ogliaro et al., 2000). The chemical reactivities of all potential HAT sites on the substrate are important for evaluating the selectivity of C–H hydroxylation (Cruciani et al., 2013; Kirchmair et al., 2015; Bonomo et al., 2017b). It has been extensively reported that DFT with dispersion corrections can correctly predict the energy barriers of C–H hydroxylation mediated by CYP enzymes (Lonsdale et al., 2010; Rydberg et al., 2014; Bonomo et al., 2017a).

In this study, DFT calculations were first performed to explore the reactivity profile for the last four carbon-atom sites of the VK1 aliphatic chain. These four sites are denoted as the ω, ω-1, ω-2 (containing ω-2R and ω-2S), and ω-3 (containing ω-3R and ω-3S) sites, respectively (**Figure 1B**). Two conformers of the isohexane with difference only in the position of the trimmed carbon atom were considered for the DFT calculations (**Figure 1B**).

The estimated highest energy-barriers are at the ω sites, ranging from 51.8 to 59.4 kJ/mol, whereas the ω-1 site has the lowest energy-barrier with about 36 kJ/mol (**Table 1**). These values are in the range for normal aliphatic hydrogen abstraction (Olsen et al., 2006) and in accordance with the order of the C–H bond strength, showing that the calculations are adequate. However, the previous experimental data showed that the oxidation of VK1 catalyzed by CYP4F2 yields ω-hydroxylated products (McDonald et al., 2009). The QM calculation results are thus inconsistent with the experimental observation.

**Table 1:** Activation energies for hydrogen abstractions in the studied sites (kJ/mol).

Sites	Conformer 1	Conformer 2
ω	54.70	51.78
^a^ω′	56.62	/
^a^ω″	59.44	/
ω-1	36.17	35.48
ω-2R	42.91	37.56
ω-2S	42.46	37.90
ω-3R	45.13	36.54
ω-3S	44.94	39.50


*^*a*^ω′ and ω″ represent the other two hydrogen atoms at the same ω site.*

**FIGURE 2:**
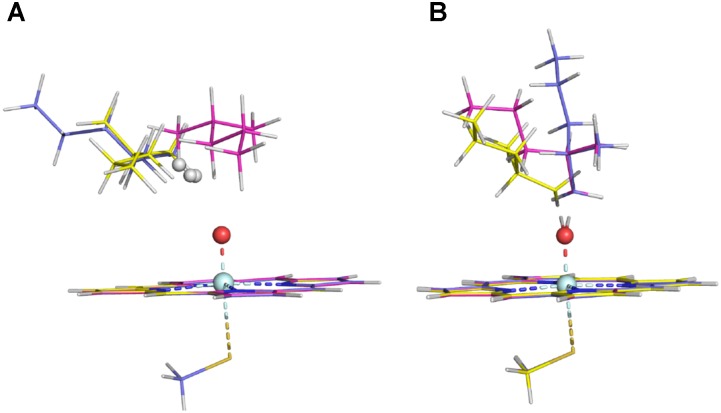
RC structures **(A)** and TS structures **(B)** in the three ω sites of Conformer 1 (the ω, ω′, and ω″ structures are colored in magenta, slate, and yellow, respectively).

The energy barriers for all the sites in conformer 2 are about 1 to 8 kJ/mol lower than those in conformer 1. We noticed that all the reactant complexes (RCs) of conformer 2 are higher in energy than those of conformer 1 and the transitional states (TSs) differ a little in the conformers 1 and 2 (**Supplementary Table S3**). This difference is likely caused by the less attractive van der Waals (vdW) interaction between isohexane and the heme plane in conformer 2 where isohexane is more perpendicular to the heme (**Supplementary Tables S4, S5** and **Supplementary Figure S2**) (Lonsdale et al., 2010; Bonomo et al., 2017b). All TS geometries are more perpendicular to the heme than the RC geometries (**Supplementary Figures S2, S3**), indicating that the attractive vdW interaction becomes repulsive when the substrate is closer to the oxo-iron. In the case of the abstraction of the hydrogen atoms at the ω site (denoted as ω, ω′, and ω″, respectively) of conformer 1, different orientations of the substrate (**Figure 2** and **Supplementary Table S3**) also result in different vdW interactions between Cpd I and the substrate, leading to different energy barriers (**Table 1**).

The QM calculations based only on the truncated Cpd I and substrate have validated the consistence between the reactivity profile of each site and the C–H bond order. However, as discussed above, the substrate conformation and relative position between Cpd I and the substrate can slightly affect the energy barrier for hydrogen abstraction due to the difference in the vdW interactions. When the dispersion correction was ignored, the difference in the energy barriers for the two conformers and that in the three ω sites of conformer 1 became smaller (**Supplementary Table S4**). These results imply that in such a small system, the dispersion interaction is important for the energy barrier prediction (Lonsdale *i*, 2010). However, the effect of vdW interaction between the substrate and protein on the hydrogen abstraction remains unclear. To understand the role of the enzymatic environment in the ω-hydroxylation of VK1, we further constructed the homology model of CYP4F2 and examined the accessibility profile of VK1.

### Construction of the CYP4F2 Model

Up to now, only one structure is available for mammalian CYP4 enzymes, which is crystalized from the rabbit CYP4B1. The identity and similarity between the sequences of CYP4F2 and CYP4B1 are 42.1% and 56.4%, respectively. The first 49 residues, which comprise the membrane embedded domain, were trimmed after the sequence alignment. All 500 Modeller outputs were evaluated by using the DOPE score. The best model (denoted as mod268) has the score of -61,956, which is close to that of the template (-65,528). Mod268 was further assessed by the VERIFY_3D and ERRAT modules in SAVES. The result showed that 88.2% of the mod268 residues have scores higher than 0.2 in VERIFY_3D, and the overall quality factor is 82.9 in ERRAT. The superposed RMSD value of the backbone atoms between mod268 and the template CYP4B1 is 0.3 Å. These suggested that mod268 has a good quality from the geometric evaluation. MD simulations were then carried out to examine the stability of the constructed homology model CYP4F2.

### MD Refinement of the mod268 Model

Unlike the enzymes of other CYP families, there is a covalent bond formed between the heme moiety and the side chain of the glutamic acid in helix I in most CYP family 4 enzymes. In CYP4F2, this special glutamic acid is Glu328, which is located in the middle of helix I. Since force field parameters for the covalent Glu328-heme moiety are currently not available, we first derived the parameters based on the CHARMM force field rules. We modified the existing heme parameters in the CHARMM 36 force field, in which a hydrogen of heme’s 5-methyl group was deleted and the force field parameters for the covalent bond were taken from those of similar residues in the force field (**Supplementary Tables S1, S2** and **Supplementary Figure S1**). The force field parameters were then tested on the CYP4B1 crystal structure. Three 100-ns MD simulations with random initial velocities were conducted to evaluate if the force field parameters were suitable for modeling the covalent Glu328-heme moiety. From the distribution of the OE–CMD bond length and the RMSD values of the heme, ligand, and backbone atoms of the protein, the derived parameters appear to be appropriate for use in modeling the CYP4 enzymes (**Supplementary Figure S4**).

**FIGURE 3:**
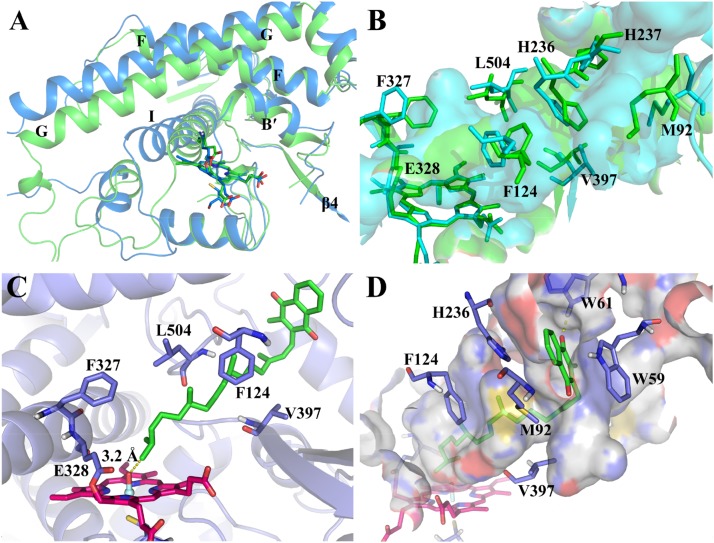
**(A)** Superposition of the major cluster of the CYP4F2 homology model after 100-ns MD simulation (marine) and the initial model (green); **(B)** Comparison of the active site residues in the MD conformation (cyan) and those in the initial model (green); **(C)** The top-1 binding mode in the inner binding pocket predicted by docking; **(D)** The top-1 binding mode in the outer binding pocket predicted by docking.

By using the derived force field parameters, three independent 100-ns MD simulations were then performed for the CYP4F2 model (mod268). The representative structure from the largest cluster in the three runs was selected for analysis and subsequent molecular docking. On the whole, the mainchain of mod268 retains all the secondary structure features of P450s and exhibits some displacements as compared to the initial structure (**Figure 3A**). The most obvious conformational changes occurred in the loops between helices G and H as well as between helices H and I, where the corresponding sequences cannot be well aligned to the template (**Supplementary Figure S5**). This region is also highly flexible and its conformation varies in different isoforms of P450s. The backbone atoms of the active site residues were also found to have slight displacement. For example, the G helix shifted upward after the MD simulations and the β4 sheet moved a little away from the active site (**Figure 3B**).

We further examined the conformational changes of the active site residues. The narrowed and slot-like topology of the active site was kept during the MD simulations, which is similar to the active site topology in the initial model (**Figure 3B**). However, the active site of the MD refined model has more branches (the cyan surface in **Figure 3B**). Phe327, which was found to be the roof the active site region near the heme (Hsu et al., 2017), shifted upward. Val397 in the turn of the β4 sheet also moved a bit outward due to the movement of the β4 sheet. The conformational changes of Phe327 and Val397 appeared to broaden the bottom of the active site. In the middle part of the active site, the displacement of Phe124, Leu504, and His236 makes this region more narrowed as compared to the initial model. The side chains of His237 and Met92 underwent larger displacement than the above residues and expanded the active site to the region near the surface of the enzyme.

### Binding Mode of VK1 With CYP4F2 by Docking

Based on the MD refined structure, the binding mode of VK1 with CYP4F2 was predicted by molecular docking. The docking results showed that the binding pose with the ω site closest to the oxo-iron has higher docking scores than other poses. The chemscore of this pose is 56.1. The distance between the ω site and the oxo-iron is 3.2 Å. There is a bottleneck, which is comprised by part of the C-terminal loop and B’ helix, limits VK1 to adopt an extended conformation (**Figure 3C**). Though the side chain of Phe327 moved upward during the MD simulation (**Figure 3B**), it together with Glu328 blocked the aliphatic chain from moving into the region close to the B-C loop. In the outer binding pocket of CYP4F2, the napthoquinone group of VK1 interacts with the residues in the loop preceding helix A (Trp59 and Trp61), the β1–β2 loop (Met92), and the turn of helix F (His236) (**Figure 3D**). Because the top five poses shared similar binding modes, only the top 1 pose was chosen for further MD simulations.

### MD Simulations on the CYP4F2-VK1 Complex

MD simulations have been widely used to evaluate the accessibility profile of substrates binding to P450s (Moors et al., 2011; Kingsley et al., 2015; Li et al., 2016; Bonomo et al., 2017a). Here, three 400-ns MD simulations were conducted for the docked VK1-CYP4F2 complex (see **Supplementary Figure S6A** for the RMSD of VK1). For each MD simulation, 200,000 frames were collected and analyzed. In most of the frames, the ω hydrogen atoms were found to be closest to the Fe-oxo atom.

**FIGURE 4:**
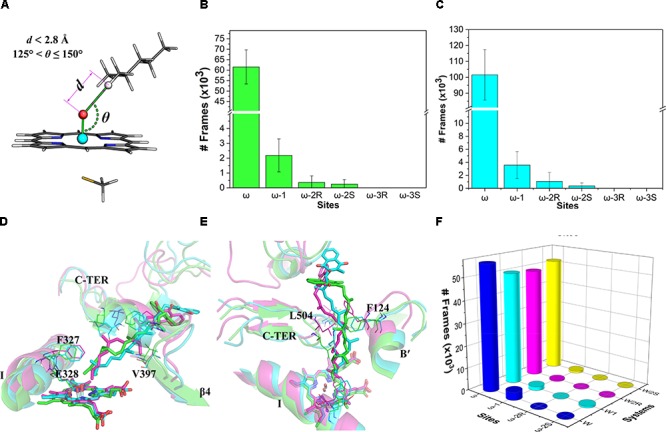
**(A)** Illustration of the distance and angle indices used for evaluating the accessibility profile of VK1; **(B)** snapshot frequency analysis for the sites fulfilling the distance and angle criteria; **(C)** snapshot frequency analysis for the sites fulfilling only the distance criteria; **(D)** superposition of the major clusters during 200–400 ns in the three independent MD simulations (side view); **(E)** Superposition of the major clusters during 200–400 ns in the three independent MD simulations (top view); **(F)** Snapshot frequency analysis for the sites fulfilling the distance and angle criteria in the W, W1, W2R, and W2S systems.

The distance (*d*) between the hydrogen atom on a potential site (ω to ω-3) and the oxo atom as well as the angle (*𝜃*) of the hydrogen, oxo, and iron atoms were measured for the ω, ω-1, ω-2, and ω-3 sites (**Figure 4A**). These two indices were used to assess the accessibility of the potential oxidation sites of VK1. The values of the indices *d* and *𝜃* were referred to the optimized RC geometries (**Supplementary Tables S5, S6**). This is because the hydrogen-oxo distance in the TS geometry is generally too short to be modeled by a conventional force field. Therefore, we adopted the following criteria for the assessment: (a) the attached hydrogen atom being the closest one to the oxo atom (i.e., with the lowest *d* value); (b) *d* ≤ 2.8 Å; and (c) 125° ≤*𝜃* ≤ 150° (**Figure 4A**). About 30% of snapshots from the MD simulations were found to satisfy the criteria. The number of snapshots with the ω site satisfying the criteria (about 92%) is much more than those of the other sites (**Figure 4B**), illustrating that the active site residues tend to orient the ω site to the oxo-iron. By comparison, none of the snapshots satisfies the distance and angle criteria for the ω-3 site. If only the distance criteria is considered for the assessment (Dubey et al., 2016), the ω hydrogen atoms are still closer to the oxo atom than other sites’ hydrogen atoms (**Figure 4C**). These results demonstrate that the oxo-iron is most accessible to the ω site when VK1 binds to the CYP4F2 active site, though the reactivity of the ω site is lower than those of the other sites.

We also analyze in detail the binding mode of VK1 in the CYP4F2 active site after the MD simulations. Although the aliphatic chain of VK1 is highly flexible, it shares a similar binding mode in the three representative structures, with the ω site pointing to the Fe-oxo atom (**Figure 4D**). Furthermore, the active site residues near the heme were relatively stable during the MD simulations (**Supplementary Figure S7**), and the RMSF values for Phe124, Phe327, Glu328, Val397, and Leu504 are lower than 2 Å, which makes the oxo-iron inaccessible to the other sites of VK1. The middle moiety of VK1 did not vary significantly, while the napthoquinone group of VK1 adopted different binding orientations (**Figure 4E**). The hydrogen bond between VK1 and Trp61 formed in the docking pose disappeared after MD simulations. The napthoquinone group of VK1 formed π–π interactions with Trp59, Phe60, Trp61, and Trp91.

To further understand the effect of the active site residues on the accessibility profile of VK1, we conducted four additional 400-ns simulations with the initial configurations favoring the hydroxylation of ω, ω-1, ω-2R, and ω-2S sites (denoted as W, W1, W2R, and W2S systems, see **Supplementary Figure S6B** for the RMSD of VK1). The initial geometries of these systems were taken from the representative structures, which were chosen based on conformation clustering of VK1 in earlier MD simulations using the *k-means* algorithm implemented in the MMTSB toolkit (Feig et al., 2004). The distance and angle analysis revealed that the binding mode in the W system was the same as in the previous 400-ns MD simulations, whereas in the W1, W2R, and W2S systems, the binding modes changed to favor the ω-hydroxylation (**Figure 4F**).

### Reaction Barriers Predicted by ONIOM Calculations

**FIGURE 5:**
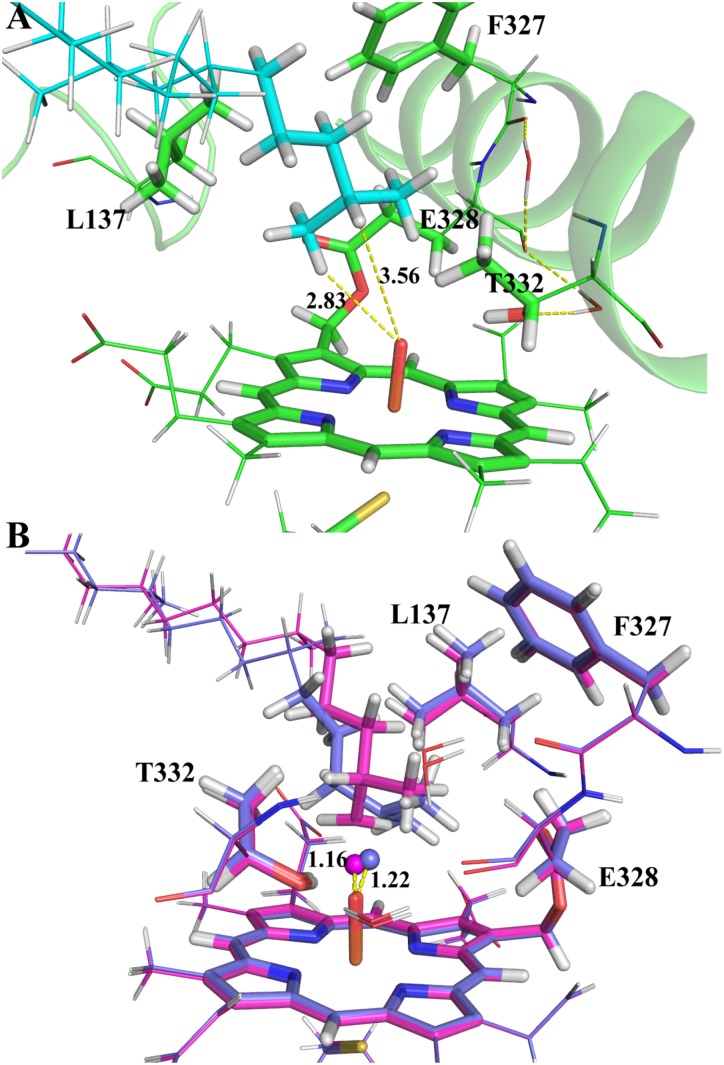
The ONIOM optimized geometries: **(A)** the RC species; **(B)** the TS species of ω (magenta) and ω-1 (marine blue) systems. The atoms in the QM region and the abstracted hydrogen atoms are represented in stick and spheres, respectively.

Though MD simulations show that the ω site of VK1 is more accessible to the oxo-iron than the ω-1 site, it is not clear how the accessibility profile affects the hydrogen activation barrier in the enzyme environment. We thus compared the activation barriers of the ω and ω-1 systems by ONIOM calculations. Because more than 90% of MD snapshots (**Figure 4**) support the ω-hydroxylation of VK1, we just chose one snapshot for the QM/MM calculation, in which the QM part of VK1 has the conformer 1 conformation. We also found that conformer 1 occupies most of all the snapshots satisfying the criteria mentioned above (see **Supplementary Figure S8**). As shown in **Figure 5A**, the distances between the oxo atom and the ω as well as ω-1 sites are 2.83 Å and 3.56 Å, respectively. The ω-2 and other sites were not considered due to the long distance between their attached hydrogen atoms and the oxo atom (more than 4 Å). Flexible scanning on such a long distance is difficult to converge and might be unreliable for predicting the activation energy of hydrogen abstraction (Dubey et al., 2016).

By explicitly including the enzyme environment, the predicted activation barriers for the ω and ω-1 are 64.6 and 69.4 kJ/mol, respectively. The ONIOM result successfully explains the selectivity of the VK1 hydroxylation by CYP4F2. The hydrogen abstraction barriers at ω and ω-1 sites are higher than those from the QM calculations (**Table 1**), which might be attributed to the narrowed protein surrounding area near the oxo moiety of Cpd I. During the flexible scanning (**Supplementary Figures S9, S10**), the substrate changed to a different orientation for exposing the tertiary hydrogen of the ω-1 site to the oxo atom. Leu137 and Thr332 underwent displacement due to the conformational shift of VK1 in the ω-1 system, resulting in a longer hydrogen-oxo distance (1.22 Å vs. 1.16 Å) and larger hydrogen-oxo-iron angle (128.3° vs. 119.8°) than those in the ω system (**Figure 5B**).

In summary, the MD simulations provide compelling evidence to support that the ω site of VK1 is the most accessible site to the Cpd I oxo atom in the active site of CYP4F2. The ONIOM calculations further proved that the accessibility profile is more important than the reactivity profile for the selectivity of VK1 hydroxylation.

## Conclusion

In this study, the ω-hydroxylation of VK1 catalyzed by CYP4F2 has been investigated in detail by different computational methods. The simple QM calculations indicated that the hydrogen abstraction barriers complied with the order of the C–H bond strength, but could not explain the hydroxylation selectivity.

The accessibility profile of VK1 approaching the iron-oxo atom was further evaluated by MD simulations based on the homology model of CYP4F2. The initial binding mode of VK1 was obtained by molecular docking. Three independent 400-ns MD simulations demonstrated that the ω-site hydrogen atoms were most accessible to the Fe-oxo atom in the catalytic center of CYP4F2. In addition, the MD simulations revealed the important roles of Phe124, Phe327, Glu328, Val368, and Leu504 in the binding of VK1 to the inner binding pocket of CYP4F2. The unbiased MD simulations thus disclosed how the enzyme active site residues influence the VK1 ω-hydroxylation at the atomistic level. The ONIOM calculations starting from the flexible scanning of ω and ω-1 sites clarified how the accessibility profile affects the activation barrier of hydroxylation mediated by CYP4F2. These results provide dynamic and atomic details on the ω-hydroxylation of VK1 mediated by CYP4F2, which are helpful not only for characterizing the function of CYP4F2 but also for looking into the mechanism of the ω-hydroxylation by other CYP4 enzymes.

## Author Contributions

WL, YaT, YuT, and GL designed the research. JL and HZ performed the research. JL analyzed the data. JL, WL, and YuT wrote the paper.

## Conflict of Interest Statement

The authors declare that the research was conducted in the absence of any commercial or financial relationships that could be construed as a potential conflict of interest.

## Footnotes

1 https://servicesn.mbi.ucla.edu/SAVES, accessed 2018/5/8
