# Supplementary material for: Computational Insight Into Vitamin K1 ω-Hydroxylation by Cytochrome P450 4F2
Source: Front Pharmacol. 2018 Sep 25;9:1065. doi: 10.3389/fphar.2018.01065 (PMC6167488; doi:10.3389/fphar.2018.01065)
Supplement: Supplementary file 1 [file Presentation_1.pdf]

Supplementary Material

Computational Insight into Vitamin K_1 ω-hydroxylation by Cytochrome P450 4F2

Junhao Li[1,2,#], Hongxiao Zhang[1,#], Guixia Liu[1], Yun Tang[1], Yaoquan Tu[2,*], Weihua Li[1,*]

[1]Shanghai Key Laboratory of New Drug Design, School of Pharmacy, East China University of Science and Technology, Shanghai 200237, China

[2]Department of Theoretical Chemistry and Biology, School of Engineering Sciences in Chemistry, Biotechnology and Health (CBH), KTH Royal Institute of Technology, SE-106 91 Stockholm, Sweden

# *These authors contributed equally to this work.*

*** Correspondence:**
whli@ecust.edu.cn (W. Li) or yaoquan@kth.se (Y. Tu)

Contents

Table S1 CHARMM partial atomic charges of GUU and HEME ·································(2)

Table S2 CHARMM force field parameters for the CMD–OE2 bond·····························(3)

Table S3 QM single point energies with dispersion correction······································ (4)

Table S4 QM Single point energies without dispersion correction··································(5)

Table S5 Features of optimized geometries of conformer 1···(6)

Table S6 Features of optimized geometries of conformer 2···(7)

Figure S1 Illustration of the CMD–OE2 covalent bond··(8)

Figure S2 Comparison of the RC structures of conformers 1 and 2··································(9)

Figure S3 Comparison of the TS structures of conformers 1 and 2································(10)

Figure S4 Tests of the CHARMN parameters··(11)

Figure S5 Sequences alignment of CYP4B1 and CYP4F2···(12)

Figure S6 RMSD values of VK1 in the MD simulations··(13)

Figure S7 RMSF values for all the protein residues··(14)

Figure S8 VK1's conformers in the MD simulations··(15)

Figure S9 The ONIOM potential energy surface of the ω system·····································(16)

Figure S10 The ONIOM potential energy surface of the ω-1 system·····························(17)

Supplementary Method for the force field parameterization···(18)

The AMBER force field parameters and atomic charge of Cpd I····································(20)

The coordinates of the QM geometries···(28)

The coordinates of the ONIOM QM region and the single point energies·······················(62)

Table S1. Partial atomic charges of GUU and HEME used in this work

Name	Type	GLU	GUU[a]	Name	Type	HEME[b]	HEME (this work)
CG	CT2	-0.28	-0.24	…	…	…	…
HG1	HA2	0.09	0.09	CMD	CT3	-0.27	0.05
HG2	HA2	0.09	0.09	1HMD	HA	0.09	0.09
CD	CD	0.62	0.80	2HMD	HA	0.09	0.09
OE1	OC	-0.76	-0.46	3HMD	HA	0.09	Deleted
OE2	OS	-0.76	-0.51	…	…	…	…
C	C	0.51	0.51				
O	O	-0.51	-0.51				
Total Charge		-1.00	-0.23	Total Charge		-2	-1.77

a. The glutamic acid covalently bound to the 5-methyl group of heme.

b. The partial atomic charges in the CHARMM36 force field.

Table S2. CHARMM force field parameters for the CMD–OE2 bond (Units: distance in nm; angle in degree; force constant in kJ/(mol·nm^2) for bonds and kJ/(mol·rad^2) for angles and dihedrals

Bonds							
OS	CT3	1	0.133	376560			
Angles							
CD	OS	CT3	5	109.6	334.72	0.22651	25104
OS	CT3	CPB	5	110.1	75.7	0	0
Dihedrals							
CD	OS	CT3	CPB	9	0	0	3
CD	OS	CT3	HA3	9	0	0	3
CT2	CD	OS	CT3	9	180	8.5772	2
OC	CD	OS	CT3	9	180	16.1084	2

Table S3. Single point energies with dispersion correction ("c1": conformer 1; "c2": conformer 2)

Systems	SP_TS[a]	ZPE_TS[b]	SP_RC[c]	ZPE_RC[d]	EA[e]	ZPE_EA[f]
c1_ω	-1862.72540392763	0.501088	-1862.75318423654	0.508035	72.937	54.698
c1_ω'	-1862.72454892340	0.501210	-1862.75274768052	0.507842	74.036	56.624
c1_ω"	-1862.72545676645	0.501302	-1862.75474953404	0.507956	76.908	59.438
c1_ω1	-1862.72540392763	0.501088	-1862.75318423654	0.508035	53.920	36.172
c1_ω2R	-1862.72454892340	0.501210	-1862.75274768052	0.507842	60.060	42.905
c1_ω2S	-1862.72545676645	0.501302	-1862.75474953404	0.507956	59.768	42.458
c1_ω3R	-1862.72540392763	0.501088	-1862.75318423654	0.508035	62.787	45.131
c1_ω3S	-1862.72454892340	0.501210	-1862.75274768052	0.507842	62.692	44.935
c2_ω	-1862.72545676645	0.501302	-1862.75474953404	0.507956	69.619	51.776
c2_ω1	-1862.72540392763	0.501088	-1862.75318423654	0.508035	53.022	35.481
c2_ω2R	-1862.72454892340	0.501210	-1862.75274768052	0.507842	54.526	37.563
c2_ω2S	-1862.72545676645	0.501302	-1862.75474953404	0.507956	55.350	37.899
c2_ω3R	-1862.72540392763	0.501088	-1862.75318423654	0.508035	54.436	36.536
c2_ω3S	-1862.72454892340	0.501210	-1862.75274768052	0.507842	56.879	39.504

[a]. The single point energies of the TS species
[b]. The zero-point energies of the TS species
[c]. The single point energies of the RC species
[d]. The zero-point energies of the RC species
[e]. The activation barrier without ZPE corrections
[f]. The activation barrier with ZPE corrections

Table S4. Single point energies without dispersion correction (“c1”: conformer 1; “c2”: conformer 2)

Systems	SP_TS[a]	ZPE_TS[b]	SP_RC[c]	ZPE_RC[d]	EA[e]	ZPE_EA[f]
c1_ω	-1862.56018006167	0.501088	-1862.59446298684	0.508035	90.010	71.770
c1_ω'	-1862.56060904937	0.501210	-1862.59441437320	0.507842	88.756	71.344
c1_ω''	-1862.55978769266	0.501302	-1862.59426745618	0.507956	90.527	73.057
c1_ω1	-1862.56458960430	0.501052	-1862.59451699478	0.507812	78.574	60.826
c1_ω2R	-1862.55992855277	0.501414	-1862.59429453958	0.507948	90.228	73.073
c1_ω2S	-1862.55993681198	0.501371	-1862.59418952146	0.507964	89.930	72.621
c1_ω3R	-1862.56124963283	0.501163	-1862.59434074041	0.507888	86.881	69.224
c1_ω3S	-1862.56130794440	0.501131	-1862.59432359195	0.507894	86.683	68.926
c2_ω	-1862.55896499456	0.501282	-1862.59347390768	0.508078	90.603	72.760
c2_ω1	-1862.56123032104	0.501324	-1862.59344233495	0.508005	84.573	67.032
c2_ω2R	-1862.56183953945	0.501549	-1862.59317402397	0.508010	82.269	65.305
c2_ω2S	-1862.56193126677	0.501451	-1862.59312620940	0.508098	81.902	64.451
c2_ω3R	-1862.56340314834	0.501090	-1862.59332844645	0.507908	78.569	60.668
c2_ω3S	-1862.56377142012	0.501257	-1862.59303041290	0.507875	76.819	59.444

[a]. The single point energies of the TS species
[b]. The zero-point energies of the TS species
[c]. The single point energies of the RC species
[d]. The zero-point energies of the RC species
[e]. The activation barrier without ZPE corrections
[f]. The activation barrier with ZPE corrections

Table S5. Optimized geometries of conformer 1

System	TS_H-O (Å)	TS_H-O-Fe (degree)	RC_H-O (Å)	RC_H-O-Fe (degree)
ω	1.16	118.7	2.79	150.6
ω'	1.15	118.6	2.70	138.7
ω''	1.16	120.0	2.69	139.7
ω-1	1.20	127.2	2.64	126.9
ω-2R	1.18	127.6	2.71	136.2
ω-2S	1.18	127.6	2.73	135.9
ω-3R	1.18	125.7	2.71	140.1
ω-3S	1.18	125.7	2.72	140.7

Table S6. Optimized geometries of conformer 2

System	TS_H-O (Å)	TS_H-O-Fe (degree)	RC_H-O (Å)	RC_H-O-Fe (degree)
ω	1.16	118.7	2.77	141.2
ω-1	1.20	128.5	2.62	140.8
ω-2R	1.19	122.1	2.65	127.5
ω-2S	1.19	122.4	2.73	125.1
ω-3R	1.19	120.5	2.58	136.9
ω-3S	1.19	120.7	2.61	131.4

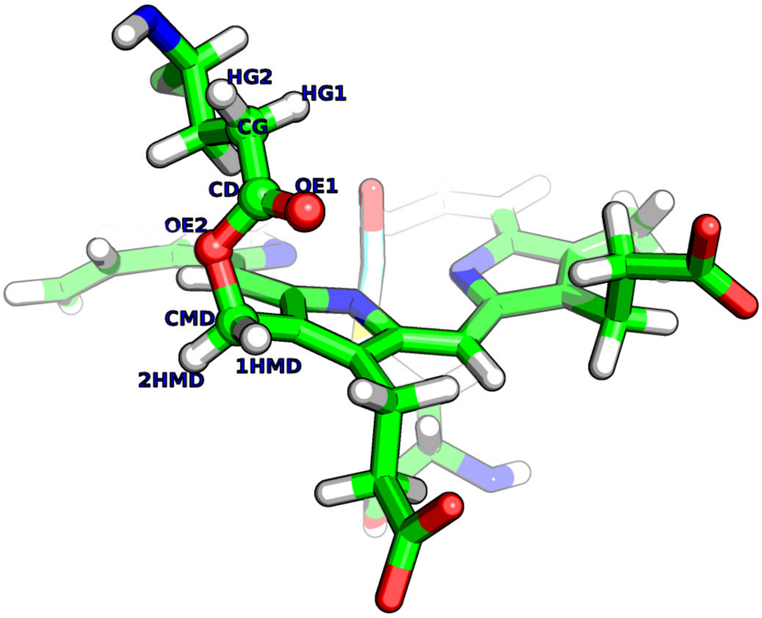

Figure S1. Illustration of the CMD–OE2 covalent bond between Heme (the Cpd I form) and Glu328 (CHARMM atom types).

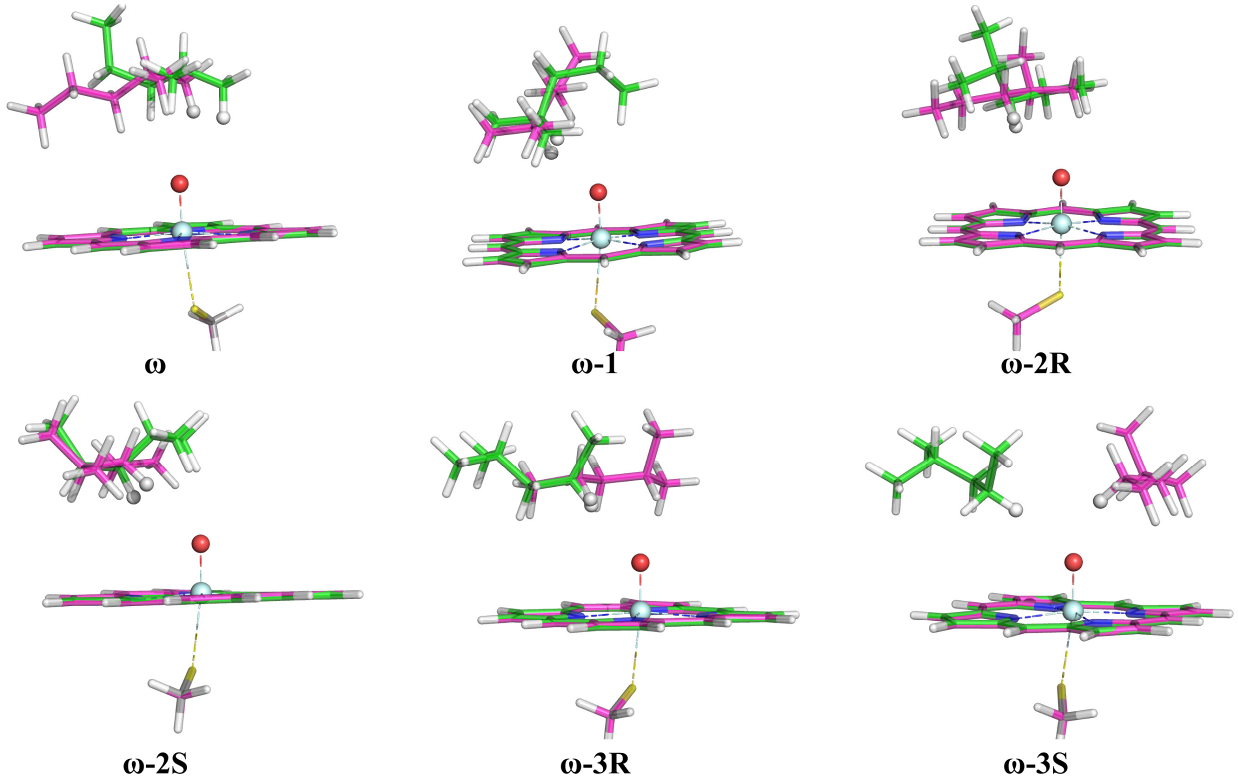

Figure S2. Comparison of the RC structures of conformers 1 and 2 at each site (conformer 1, magenta; conformer 2, green).

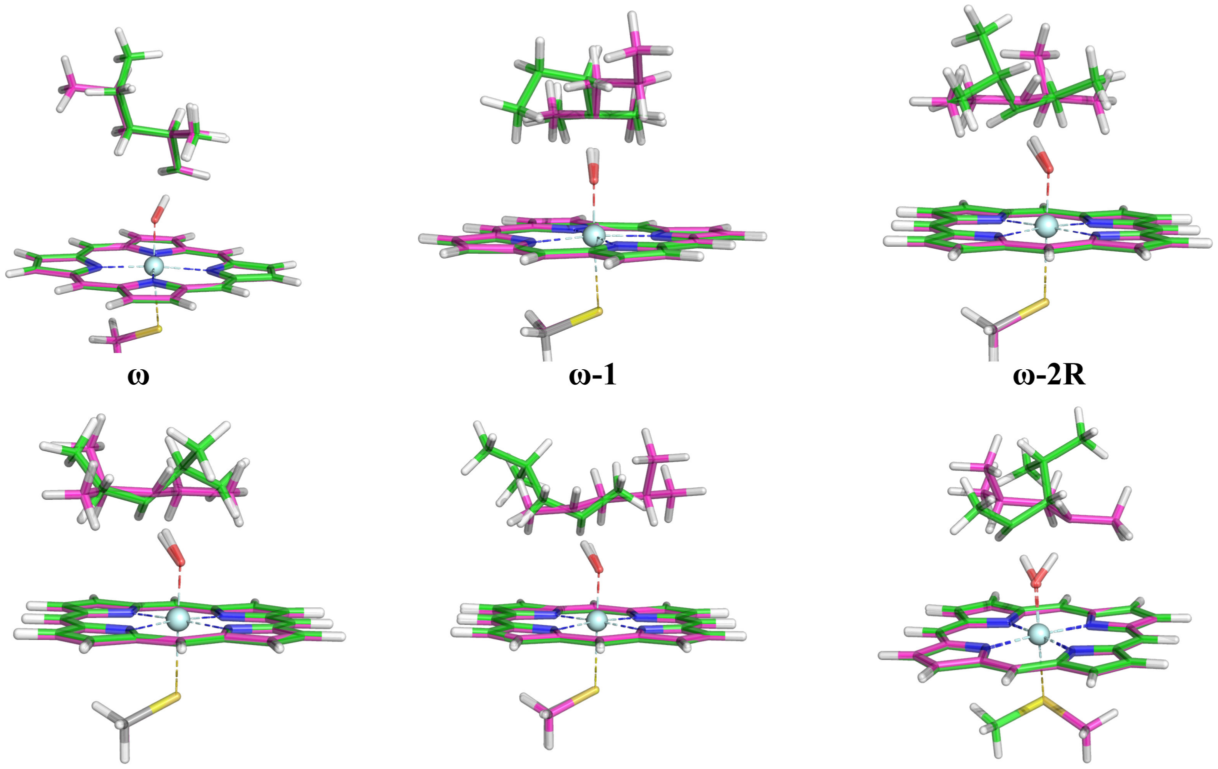

ω **ω-1** **ω-2R**

ω-2S **ω-3R** **ω-3S**

Figure S3. Comparison of the TS structures of conformers 1 and 2 at each site (conformer 1, magenta; conformer 2, green).

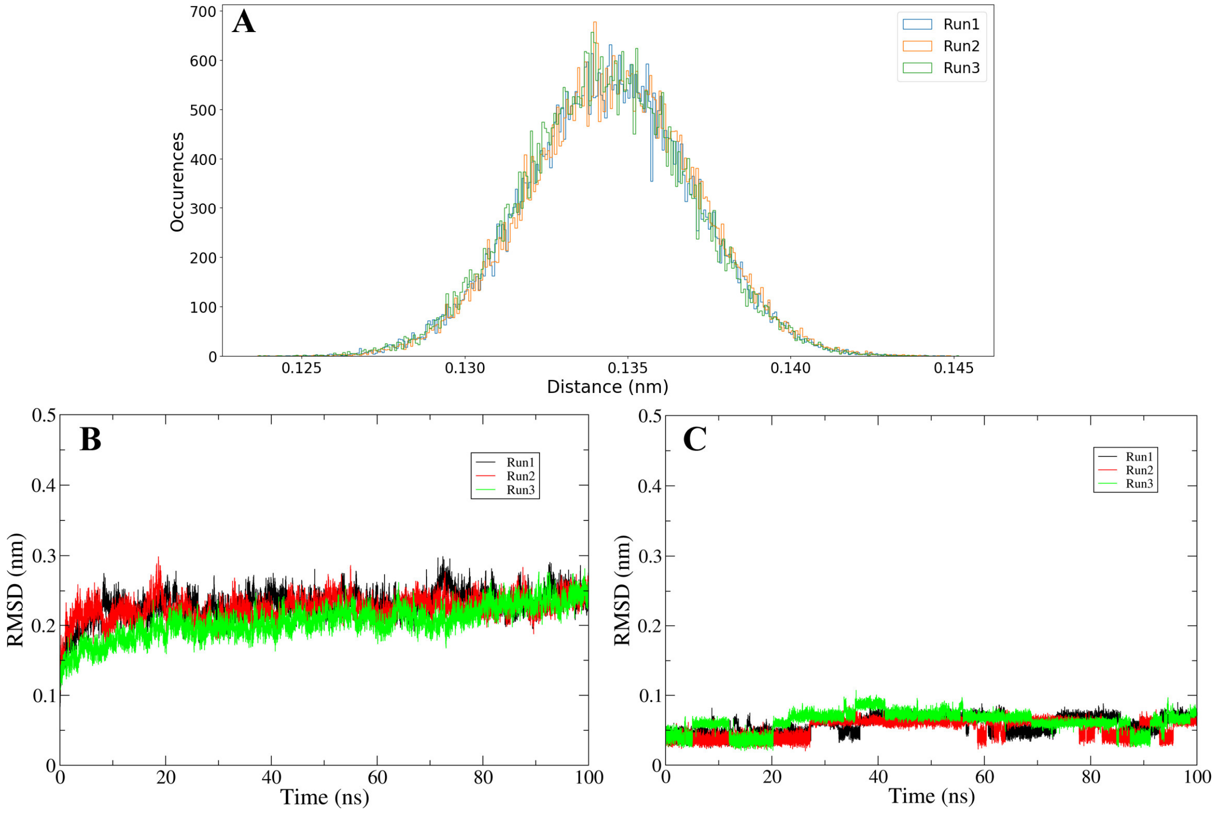

Figure S4. (A) Histogram distribution of the OE-CMD distance in the three independent 100-ns simulations of CYP4B1; (B) RMSDs of the protein backbone atoms in the three independent 100-ns simulations of CYP4B1; (C) RMSDs of the heme residue in the three independent 100-ns simulations of CYP4B1

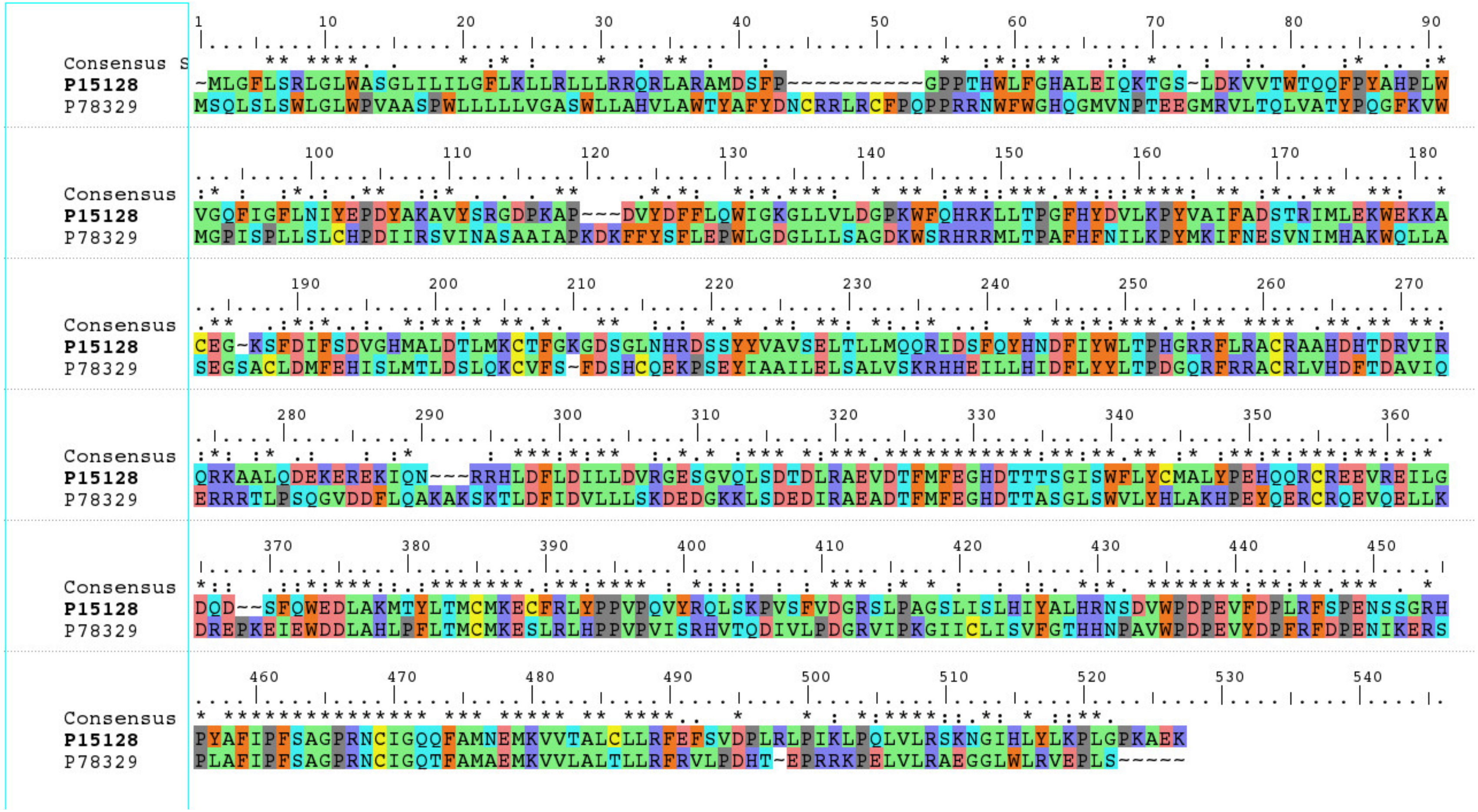

Figure S5. Sequences alignment of CYP4B1 (P15128) and CYP4F2 (P78329)

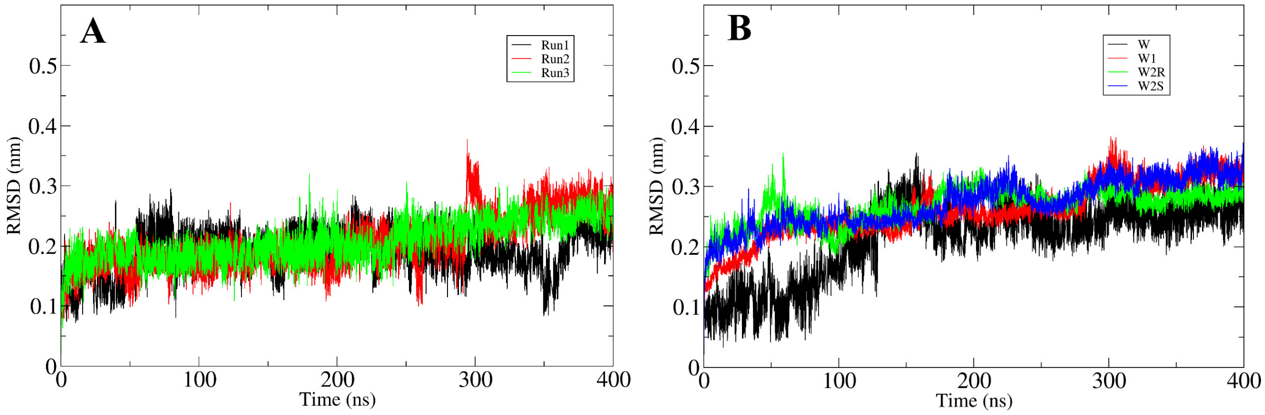

Figure S6. RMSD values of VK1 in the three independent (A) and four extended (B) MD simulations

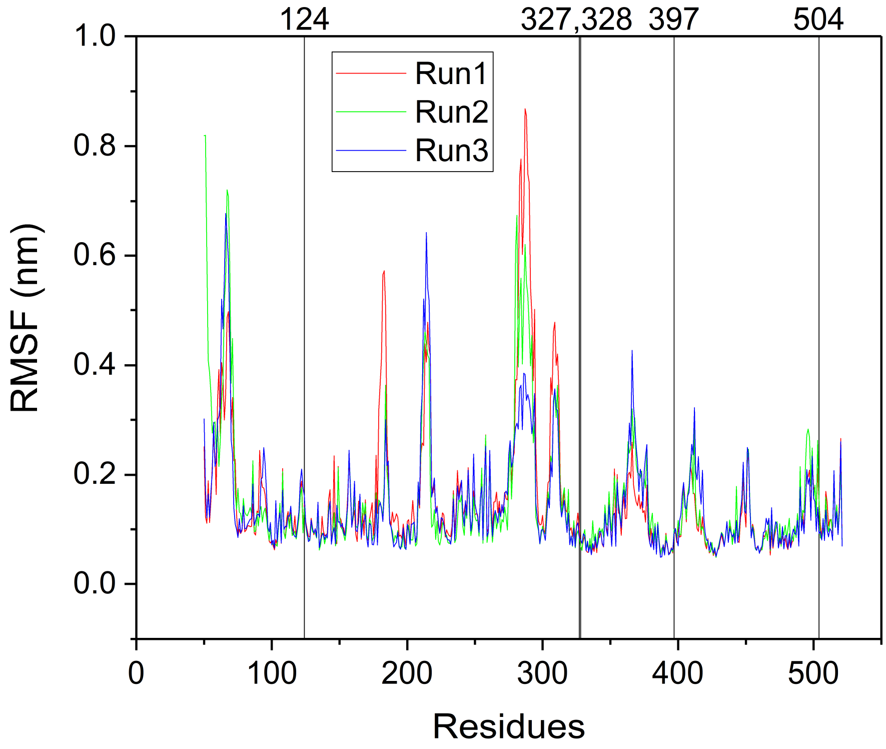

Figure S7. RMSF values for all the protein residues from the three independent 400-ns MD simulations

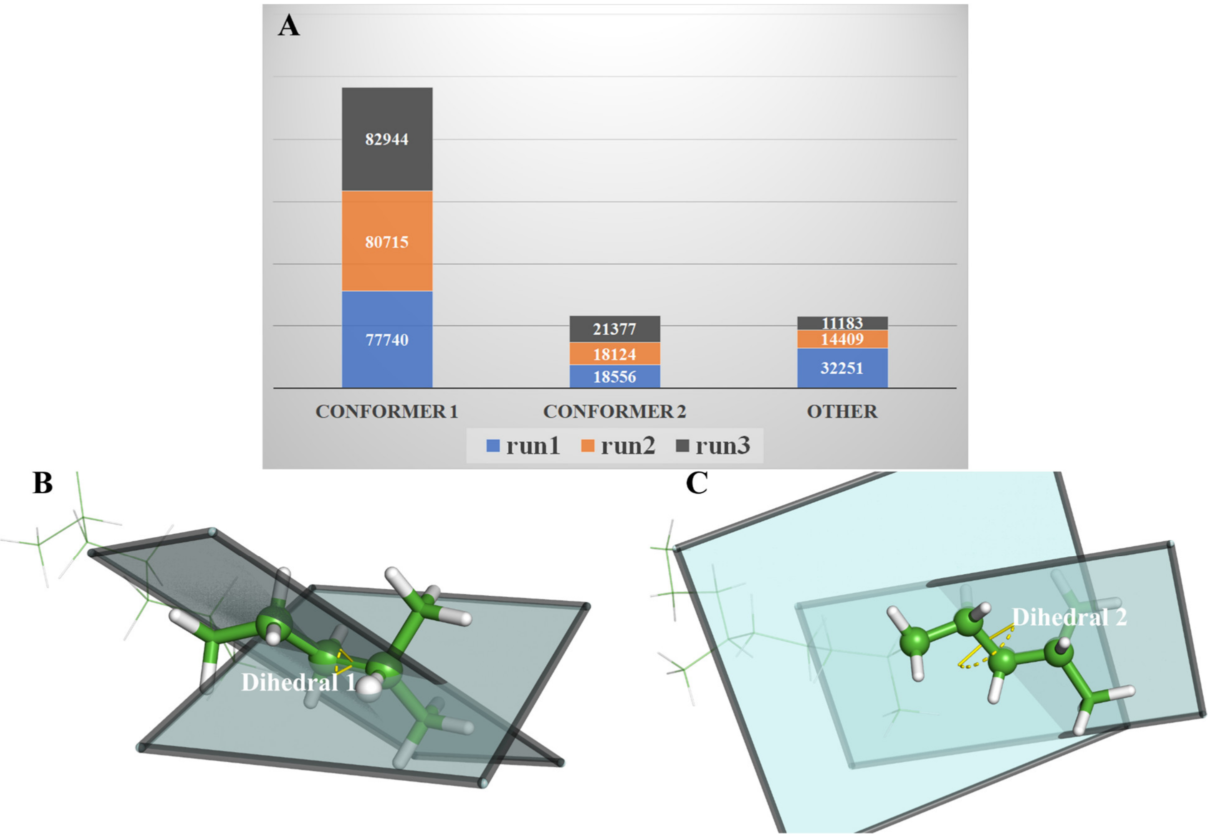

Figure S8 (A). The numbers of conformers 1 and 2 amongst the snapshots with one site of VK1 fulling the criteria mentioned in our paper. (B). and (C)., Two dihedrals (atoms showing in spheres) distinguishing the conformers 1 and 2 were used for conformation counting:

Conformer 1: dihedral 1 ranges [-90°, -30°] , [30°, 90°]

dihedral 2 ranges [-180°, -150°], [150°, 180°]

Conformer 2: dihedral 1 ranges [-90°, -30°] , [30°, 90°]

dihedral 2 ranges [-90°, -30°] , [30°, 90°]

Other: dihedrals 1 and 2 out of the ranges of conformers 1 and 2.

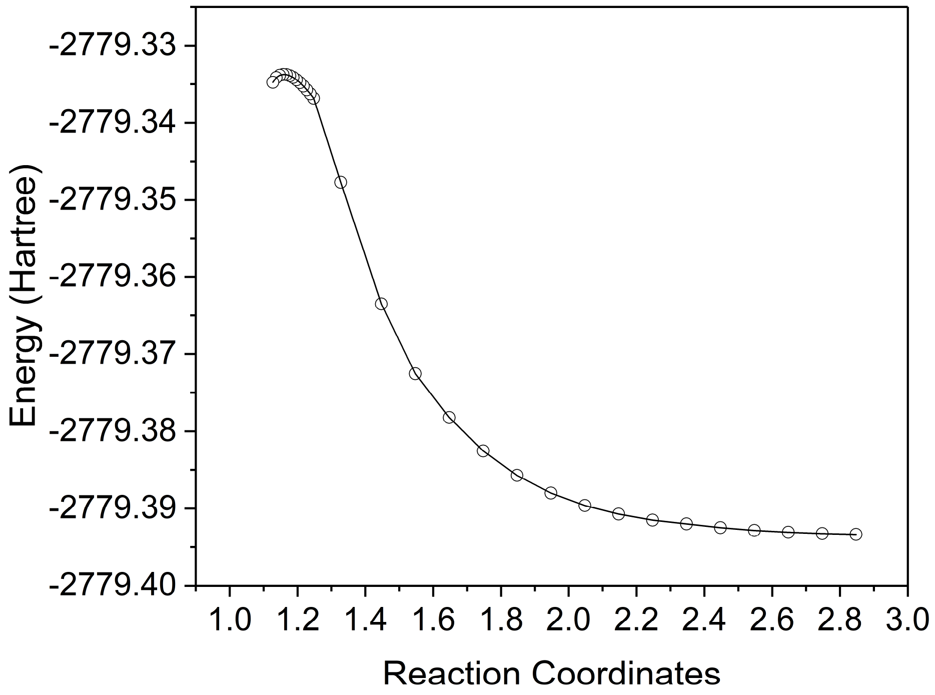

Figure S9 The ONIOM potential energy surface of the ω system along the reaction coordinates. The step size of the flexible scanning was changed from 0.1 to 0.01 Å in the ending stage.

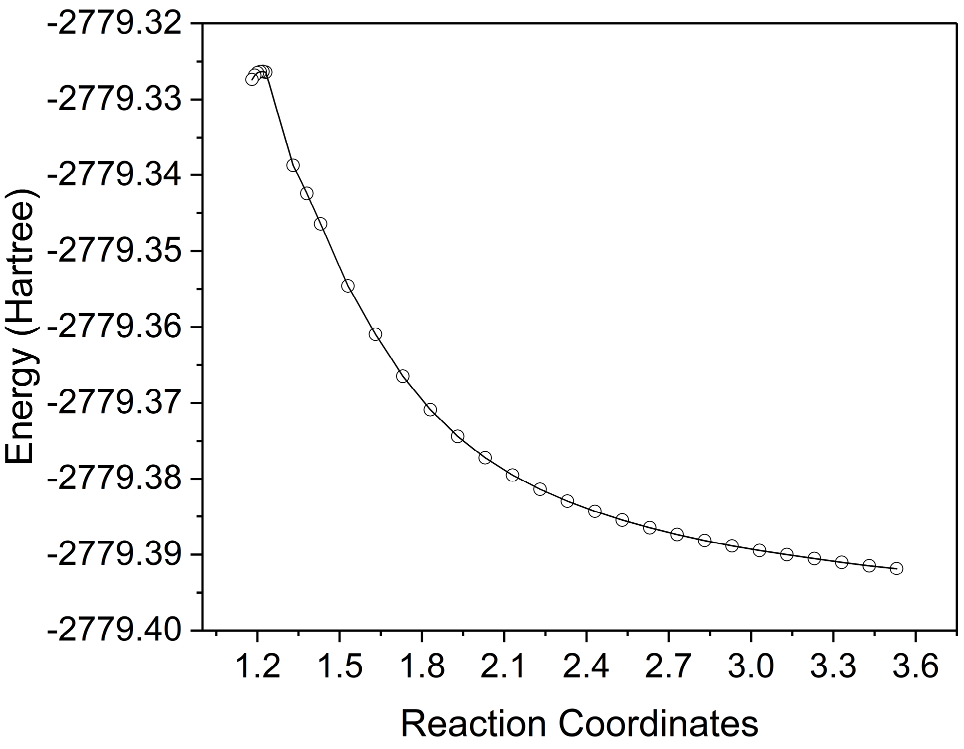

Figure S10 The ONIOM potential energy surface of the ω-1 system along the reaction coordinates. The step size of the flexible scanning was changed from 0.1 to 0.01 Å in the ending stage.

Supplementary Method

The AMBER force field was used for the MM part in the ONIOM simulations. The force field parameters regarding to the bond between Glu328 and Heme were adopted from the similar terms in the general amber force field[1] (GAFF, by searching the *gaff.dat*), while other parameters of heme was adopted from Shahrokh's work.[2] Since the charge distribution in AMBER force field is not grouped, we followed a similar procedure as on the AMBER website[3] to fit the charge of Glu328, Heme and Cys468. Briefly, the three units were extracted as a chimera unit, namely HEU, from an energy-minimized snapshot with the capped atoms added to the Glu328 and Cys468. The geometry of the HEU was then optimized at b3lyp level using the combined 6-31g(d)/LANL2DZ basis set in Gaussian 09.[4] The dihedral angles of the propionate groups of Heme and the coordinates of the C and N of the capped atoms were restricted to avoid unphysical conformations. The electrostatic potential (ESP) for the atoms of the optimized geometry was then derived at the b3lyp level using the cc-PVTZ basis set. The high spin state of the iron was adopted for both the geometry optimization and ESP fitting. The restricted ESP (RESP) charged fitting were accomplished by using the Antehcamber and xleap modules of Amber 16.[5] Finally, the GUU, HEM, and CYI residues for the new Cpd I unit were separated from the HEU by removing the capped atoms.

Reference

[1] Wang, J. , Wolf, R. M., Caldwell, J. W., Kollman, P. A. and Case, D. A. (2004), Development and testing of a general amber force field. J. Comput. Chem., 25: 1157-1174.

[2] Shahrokh, K. , Orendt, A. , Yost, G. S. and Cheatham, T. E. (2012), Quantum mechanically derived AMBER‐compatible heme parameters for various states of the cytochrome P450 catalytic cycle. J. Comput. Chem., 33: 119-133.

[3] http://ambermd.org/tutorials/advanced/tutorial1/index.htm (accessed 2018-08-17)

[4] M. J. Frisch, G. W. Trucks, H. B. Schlegel, G. E. Scuseria, M. A. Robb, J. R. Cheeseman, G. Scalmani, V. Barone, B. Mennucci, G. A. Petersson, H. Nakatsuji, M. Caricato, X. Li, H. P. Hratchian, A. F. Izmaylov, J. Bloino, G. Zheng, J. L. Sonnenberg, M. Hada, M. Ehara, K. Toyota, R. Fukuda, J. Hasegawa, M. Ishida, T. Nakajima, Y. Honda, O. Kitao, H. Nakai, T. Vreven, J. A. Montgomery, Jr., J. E. Peralta, F. Ogliaro, M. Bearpark, J. J. Heyd, E. Brothers, K. N. Kudin, V. N. Staroverov, R. Kobayashi, J. Normand, K. Raghavachari, A. Rendell, J. C. Burant, S. S. Iyengar, J. Tomasi, M. Cossi, N. Rega, J. M. Millam, M. Klene, J. E. Knox, J. B. Cross, V. Bakken, C. Adamo, J. Jaramillo, R. Gomperts, R. E. Stratmann, O. Yazyev, A. J. Austin, R. Cammi, C. Pomelli, J. W. Ochterski, R. L. Martin, K. Morokuma, V. G. Zakrzewski,

G. A. Voth, P. Salvador, J. J. Dannenberg, S. Dapprich, A. D. Daniels, Ö. Farkas, J. B. Foresman, J. V. Ortiz, J. Cioslowski, and D. J. Fox, Gaussian 09 (Gaussian, Inc., Wallingford CT, 2009).

[5] D.A. Case, I.Y. Ben-Shalom, S.R. Brozell, D.S. Cerutti, T.E. Cheatham, III, V.W.D. Cruzeiro, T.A. Darden, R.E. Duke, D. Ghoreishi, M.K. Gilson, H. Gohlke, A.W. Goetz, D. Greene, R Harris, N. Homeyer, S. Izadi, A. Kovalenko, T. Kurtzman, T.S. Lee, S. LeGrand, P. Li, C. Lin, J. Liu, T. Luchko, R. Luo, D.J. Mermelstein, K.M. Merz, Y. Miao, G. Monard, C. Nguyen, H. Nguyen, I. Omelyan, A. Onufriev, F. Pan, R. Qi, D.R. Roe, A. Roitberg, C. Sagui, S. Schott-Verdugo, J. Shen, C.L. Simmerling, J. Smith, R. Salomon-Ferrer, J. Swails, R.C. Walker, J. Wang, H. Wei, R.M. Wolf, X. Wu, L. Xiao, D.M. York and P.A. Kollman (2016), AMBER 2016, University of California, San Francisco.

The AMBER force field parameters and atomic charge of the new Cpd I units

(a) Compound I (CPDI) AMBER parameter file:

frcmod.heu2018

```
#force field for HEU, added in addition to those from Shahrokh et al (Jour Comp Chem (2011))
MASS
fe  55.85
oa  16.00

BOND
fe-nc      114.00       2.029
fe-nd      114.00       2.029
fe-SH       39.00       2.565
cg-ha      341.50       1.089 # same as ce-ha  341.5    1.089        SOURCE3
fe-oa      572.00       1.639
c3-OS      390.80       1.358 # same as c -os #ADDITIONAL4CYP4

ANGLE
nc-fe-oa    65.000     92.406 # average angle
nd-fe-oa    65.000     92.406 # average angle
SH-fe-oa     0.000    174.087 #
nc-fe-nd   239.000     89.900 # average angle
fe-nc-cc   146.000    126.651 # average angle
fe-nd-cd   146.000    126.651 # average angle
nc-fe-nc     0.000    174.731
nd-fe-nd     0.000    175.636
SH-fe-nc    48.00      87.595 # average angle
SH-fe-nd    48.00      87.595 # average angle
CT-SH-fe    39.00     105.885
cc-cc-cg    65.6      124.539 # same as cc-cc-cf    65.6      123.92    SOURCE3
cd-cd-cg    65.6      124.539 # same as cc-cc-cf    65.6      123.92    SOURCE3
nc-cc-cg    68.5      125.044 # same as cf-cf-n2    68.5      123.00    SOURCE3
nd-cd-cg    68.5      125.044 # same as cf-cf-n2    68.5      123.00    SOURCE3
ha-cc-ha    38.0      117.65  # same as ha-c2-ha    38.0      117.65    SOURCE3
ha-cd-ha    38.0      117.65  # same as ha-c2-ha    38.0      117.65    SOURCE3
cc-cg-ha    46.6      116.969 # same as cd-cd-ha    46.6      123.74    SOURCE3
cd-cg-ha    46.6      116.969 # same as cd-cd-ha    46.6      123.74    SOURCE3
cd-cg-cc    63.8      126.057 # same as ce-ce-cf    63.8      130.92    SOURCE3
OS-c3-cd    68.54     108.560 # same as c2-c3-os   #ADDITIONAL4CYP4
OS-c3-hc    51.05     108.700 # same as hc-c3-os   #ADDITIONAL4CYP4
O2-C-OS     75.32     123.250 # same as o -c -os   #ADDITIONAL4CYP4
C-OS-c3     63.28     115.980 # same as c -os-c3   #ADDITIONAL4CYP4

DIHEDRAL
cd-nd-fe-oa  1     0.000     180.000       2.000
cc-nc-fe-oa  1     0.000     180.000       2.000
CT-SH-fe-nc  1     0.00      180.000       2.000
CT-SH-fe-nd  1     0.00      180.000       2.000
CT-SH-fe-oa  1     0.00      180.000       2.000
cc-cc-c3-c3  3     0.000     180.000       2.000
cc-cc-c3-hc  3     0.000     180.000       2.000
```

```
cd-cd-c3-c3  3     0.000     180.000       2.000
cd-cd-c3-hc  3     0.000     180.000       2.000
X -cg-cd-X   4     16.00     180.000       2.000
X -cg-cc-X   4     16.00     180.000       2.000
cg-cc-nc-fe  1     0.000     180.000       2.000
cg-cd-nd-fe  1     0.000     180.000       2.000
cc-cc-nc-fe  1     0.000     180.000       2.000
cd-cd-nd-fe  1     0.000     180.000       2.000
nd-fe-nc-cc  1     0.000     180.000       2.000
nc-fe-nd-cd  1     0.000     180.000       2.000
cc-nc-fe-nc  1     0.000     180.000       2.000
cd-nd-fe-nd  1     0.000     180.000       2.000
cc-nc-fe-SH  1     0.000     180.000       2.000
cd-nd-fe-SH  1     0.000     180.000       2.000
X -c3-OS-X   3     1.150       0.000       3.000  #same as X -c3-os-X   #ADDITIONAL4CYP4

IMPROPER

NONBON
fe 1.3    0.01
oa 1.6612 0.21
```

(b) Hexa-coordinate Compound I coordinates and atomic charges

HEM.mol2

```
# HEM unit for CYP4 enzyme, Li et al (Frontiers in Pharmacology, 2018)
@<TRIPOS>MOLECULE
'HEM'
   73    80     1     0     1
SMALL
USER_CHARGES
@<TRIPOS>ATOM
   1 FE       4.912522    1.146819    6.284422 fe     1 HEM      0.3787 ****
   2 OE       5.214431    0.127962    7.532262 oa     1 HEM     -0.5135 ****
   3 NA       6.847637    1.466750    6.457438 nc     1 HEM      0.0134 ****
   4 C1A      7.856928    1.046557    5.594177 cc     1 HEM      0.0898 ****
   5 CHA      7.759893    0.067616    4.639243 cg     1 HEM     -0.2667 ****
   6 HHA      8.576002   -0.225472    3.977165 ha     1 HEM      0.2050 ****
   7 C2A      9.122758    1.686404    5.923126 cc     1 HEM     -0.2462 ****
   8 CAA     10.367540    1.395392    5.228249 c3     1 HEM      0.0300 ****
   9 CBA     10.971509    0.133314    5.827301 c3     1 HEM     -0.1721 ****
  10 CGA     12.385641   -0.464834    5.446283 c      1 HEM      0.6913 ****
  11 O1A     13.114874   -0.860988    6.407626 o      1 HEM     -0.5690 ****
  12 O2A     12.774755   -0.517898    4.266951 o      1 HEM     -0.5690 ****
  13 HBA1    10.299226   -0.730970    5.639234 h1     1 HEM      0.0627 ****
  14 HBA2    11.018490    0.292272    6.924855 h1     1 HEM      0.0627 ****
  15 HAA1    11.072359    2.251087    5.258234 hc     1 HEM      0.0462 ****
  16 HAA2    10.115489    1.158345    4.163036 hc     1 HEM      0.0462 ****
  17 C3A      8.945750    2.377584    7.057383 cc     1 HEM      0.2101 ****
  18 CMA     10.008033    3.024691    7.868576 c3     1 HEM     -0.4605 ****
  19 HMA1    10.980105    2.577591    7.588552 hc     1 HEM      0.1179 ****
  20 HMA2    10.098125    4.108101    7.625469 hc     1 HEM      0.1179 ****
  21 HMA3     9.812958    2.745622    8.921911 hc     1 HEM      0.1179 ****
  22 C4A      7.475321    2.314650    7.390486 cc     1 HEM     -0.0763 ****
  23 CHB      6.849493    3.105530    8.274301 cg     1 HEM     -0.0457 ****
  24 HHB      7.556472    3.572474    8.955265 ha     1 HEM      0.1203 ****
  25 C1B      5.507561    3.147606    8.478295 cd     1 HEM     -0.2184 ****
  26 C2B      4.969636    3.962607    9.535239 cd     1 HEM      0.1022 ****
  27 C3B      3.625455    3.580650    9.468244 cd     1 HEM     -0.0191 ****
  28 CAB      2.517657    4.070660   10.168154 cc     1 HEM     -0.0401 ****
  29 CBB      2.561719    4.904531   11.184000 cd     1 HEM     -0.4452 ****
  30 HBB1     1.675713    5.217600   11.750030 ha     1 HEM      0.1478 ****
  31 HBB2     3.592504    5.246374   11.345956 ha     1 HEM      0.1478 ****
  32 HAB      1.555999    3.670885    9.820305 ha     1 HEM      0.0897 ****
  33 CMB      5.782726    4.931597   10.376251 c3     1 HEM     -0.1811 ****
  34 HMB1     5.926718    4.550587   11.415267 hc     1 HEM      0.0476 ****
  35 HMB2     6.773825    5.195551    9.949200 hc     1 HEM      0.0476 ****
  36 HMB3     5.260743    5.918704   10.347233 hc     1 HEM      0.0476 ****
  37 NB       4.594433    2.353577    7.774224 nd     1 HEM      0.1455 ****
  38 C4B      3.427158    2.542684    8.421381 cd     1 HEM     -0.0241 ****
  39 CHC      2.290273    1.918829    8.154411 cg     1 HEM     -0.2475 ****
  40 HHC      1.415986    2.043918    8.796625 ha     1 HEM      0.1614 ****
  41 C1C      2.069255    0.985971    7.146538 cc     1 HEM     -0.1120 ****
  42 C2C      0.815092    0.325970    6.859498 cc     1 HEM      0.2532 ****
```

```
 43 C3C      1.183994   -0.738912    6.059586 cc     1 HEM    -0.1465 ****
 44 CAC      0.420104   -1.832630    5.641665 cd     1 HEM     0.0144 ****
 45 CBC     -0.883006   -2.027574    5.906687 cc     1 HEM    -0.4111 ****
 46 HBC1    -1.304111   -2.996638    5.593653 ha     1 HEM     0.1194 ****
 47 HBC2    -1.515092   -1.320391    6.461809 ha     1 HEM     0.1194 ****
 48 HAC      0.934285   -2.586977    5.033398 ha     1 HEM     0.1277 ****
 49 CMC     -0.616922    0.827046    7.200501 c3     1 HEM    -0.3525 ****
 50 HMC1    -1.147097    1.045160    6.244171 hc     1 HEM     0.0909 ****
 51 HMC2    -1.140003    0.076003    7.828531 hc     1 HEM     0.0909 ****
 52 HMC3    -0.533855    1.773382    7.772714 hc     1 HEM     0.0909 ****
 53 NC       3.123454    0.450808    6.448402 nc     1 HEM     0.2795 ****
 54 C4C      2.598576   -0.577807    5.790606 cc     1 HEM    -0.1423 ****
 55 CHD      3.295787   -1.447143    5.002332 cg     1 HEM    -0.1118 ****
 56 HHD      2.984827   -2.455829    4.730383 ha     1 HEM     0.1473 ****
 57 C1D      4.610472   -1.343239    4.718419 cd     1 HEM    -0.0927 ****
 58 ND       5.399281   -0.369571    5.100324 nd     1 HEM     0.1397 ****
 59 C4D      6.605207   -0.657614    4.529361 cd     1 HEM    -0.0077 ****
 60 C3D      6.592142   -1.928575    3.827370 cd     1 HEM     0.0688 ****
 61 CAD      7.601127   -2.466641    2.889353 c3     1 HEM    -0.1655 ****
 62 CBD      7.946098   -1.590845    1.694607 c3     1 HEM    -0.0858 ****
 63 CGD      8.871205   -2.246005    0.595434 c      1 HEM     0.6844 ****
 64 O1D      9.969011   -2.755982    0.976364 o      1 HEM    -0.5670 ****
 65 O2D      8.498115   -2.154951   -0.609862 o      1 HEM    -0.5670 ****
 66 HBD1     8.524237   -0.690726    1.990651 h1     1 HEM     0.0431 ****
 67 HBD2     6.996703   -1.329683    1.184387 h1     1 HEM     0.0431 ****
 68 HAD1     7.335978   -3.478960    2.522218 hc     1 HEM     0.0703 ****
 69 HAD2     8.511832   -2.646650    3.505151 hc     1 HEM     0.0703 ****
 70 C2D      5.363815   -2.395637    4.004405 cd     1 HEM    -0.1179 ****
 71 CMD      4.841758   -3.797573    3.626409 c3     1 HEM     0.2374 ****
 72 HMD1     5.682085   -4.459890    3.316275 hc     1 HEM     0.0307 ****
 73 HMD2     4.042731   -3.743521    2.856374 hc     1 HEM     0.0307 ****
@<TRIPOS>BOND
     1    71    72 1
     2    71    73 1
     3    70    71 1
     4    63    64 1
     5    63    65 1
     6    62    63 1
     7    62    66 1
     8    62    67 1
     9    61    62 1
    10    61    68 1
    11    61    69 1
    12    60    61 1
    13    60    70 1
    14    59    60 1
    15    58    59 1
    16    57    58 1
    17    57    70 1
    18    55    56 1
    19    55    57 1
    20    54    55 1
    21    53    54 1
```

22	49	50	1
23	49	51	1
24	49	52	1
25	45	46	1
26	45	47	1
27	44	45	1
28	44	48	1
29	43	44	1
30	43	54	1
31	42	43	1
32	42	49	1
33	41	42	1
34	41	53	1
35	39	40	1
36	39	41	1
37	38	39	1
38	37	38	1
39	33	34	1
40	33	35	1
41	33	36	1
42	29	30	1
43	29	31	1
44	28	29	1
45	28	32	1
46	27	28	1
47	27	38	1
48	26	27	1
49	26	33	1
50	25	26	1
51	25	37	1
52	23	24	1
53	23	25	1
54	22	23	1
55	18	19	1
56	18	20	1
57	18	21	1
58	17	18	1
59	17	22	1
60	10	11	1
61	10	12	1
62	9	10	1
63	9	13	1
64	9	14	1
65	8	9	1
66	8	15	1
67	8	16	1
68	7	8	1
69	7	17	1
70	5	6	1
71	5	59	1
72	4	5	1
73	4	7	1
74	3	4	1

```
    75      3     22 1
    76      1      2 1
    77      1      3 1
    78      1     37 1
    79      1     53 1
    80      1     58 1
@<TRIPOS>SUBSTRUCTURE
     1  HEM              1 ****               0 ****  ****
```

(c) Coordinates and atomic charge of the Cystine of Cpd I

CYI.mol2

```
# CYS unit for CYP4 enzyme, Li et al(Frontiers in Pharmacology, 2018)
@<TRIPOS>MOLECULE
CYI
   10     9     1     0     1
SMALL
USER_CHARGES
@<TRIPOS>ATOM
  1 N        7.464258    3.436989    1.911955 N      1 CYI    -0.8001 ****
  2 H        7.909403    4.149183    2.480127 H      1 CYI     0.2983 ****
  3 CA       6.237018    2.796924    2.354007 CT     1 CYI     0.7975 ****
  4 HA       6.491991    1.741912    2.474016 H1     1 CYI    -0.1000 ****
  5 CB       5.815995    3.431009    3.703109 CT     1 CYI    -0.0238 ****
  6 HB2      6.741205    3.327953    4.300271 HC     1 CYI     0.0555 ****
  7 HB3      5.782020    4.512455    3.494006 HC     1 CYI     0.0555 ****
  8 SG       4.255563    2.783919    4.422291 SH     1 CYI    -0.6929 ****
  9 C        5.106350    2.816951    1.301290 C      1 CYI     0.2946 ****
 10 O        4.838398    3.854909    0.704318 O      1 CYI    -0.5250 ****
@<TRIPOS>BOND
     1     9    10 1
     2     5     6 1
     3     5     7 1
     4     5     8 1
     5     3     4 1
     6     3     5 1
     7     3     9 1
     8     1     2 1
     9     1     3 1
@<TRIPOS>SUBSTRUCTURE
      1  CYI              1 ****               0 ****  ****
```

(d) Coordinates and atomic charge of the Glutamic acid of Cpd I

GUU.mol2

```
# GUU unit for CYP4 enzyme, Li et al (Frontiers in Pharmacology, 2018)
@<TRIPOS>MOLECULE
GUU
   15    14     1     0     1
SMALL
USER_CHARGES
@<TRIPOS>ATOM
  1 N        2.735699   -5.630093    9.447980 N      1 GUU    -0.6100 ****
  2 H        2.491725   -6.219963    8.669148 H      1 GUU     0.2965 ****
  3 CA       3.061687   -4.267454    9.063070 CT     1 GUU     0.3478 ****
  4 HA       3.953372   -3.938620    9.600871 H1     1 GUU     0.0448 ****
  5 CB       3.314677   -4.281484    7.547099 CT     1 GUU    -0.1630 ****
  6 HB2      2.430328   -4.606550    6.967880 HC     1 GUU     0.0562 ****
  7 HB3      3.665618   -3.252843    7.298176 HC     1 GUU     0.0562 ****
  8 CG       4.538580   -5.101524    7.153117 CT     1 GUU    -0.1164 ****
  9 HG2      5.325266   -5.140557    7.933811 HC     1 GUU     0.0583 ****
 10 HG3      4.341570   -6.174225    6.952170 HC     1 GUU     0.0583 ****
 11 CD       5.214395   -4.621705    5.844511 C      1 GUU     0.5219 ****
 12 OE1      6.493310   -4.640781    5.629522 O2     1 GUU    -0.5083 ****
 13 OE2      4.357600   -4.382706    4.855732 OS     1 GUU    -0.3264 ****
 14 C        1.914530   -3.267213    9.340122 C      1 GUU     0.4368 ****
 15 O        2.155502   -2.065637    9.375101 O      1 GUU    -0.5375 ****
@<TRIPOS>BOND
     1     1     2 1
     2     1     3 1
     3     3     4 1
     4     3     5 1
     5     3    14 1
     6     5     6 1
     7     5     7 1
     8     5     8 1
     9     8     9 1
    10     8    10 1
    11     8    11 1
    12    11    12 1
    13    11    13 1
    14    14    15 1
@<TRIPOS>SUBSTRUCTURE
      1  GUU              1 ****               0 ****  ****
```

The coordinates of the QM optimized geometries

RCs of conformer 1:

ω

O	-0.629809	-0.732203	0.536817
Fe	-0.226634	0.436275	-0.515018
N	0.435067	-0.856182	-1.914628
N	1.653113	0.620579	0.189014
N	-0.822600	1.942757	0.691056
N	-2.023029	0.498271	-1.436885
C	-0.311065	-1.475800	-2.881663
C	0.488863	-2.444959	-3.590874
C	1.730139	-2.409724	-3.033599
C	1.686783	-1.418748	-1.985752
C	2.741315	-0.130225	-0.168410
C	3.880641	0.216647	0.646922
C	3.464473	1.189309	1.503832
C	2.071493	1.428686	1.211853
C	-0.069210	2.572433	1.651005
C	-0.884057	3.500746	2.397280
C	-2.141833	3.415527	1.886043
C	-2.091760	2.442847	0.821039
C	-3.138182	1.199922	-1.028049
C	-4.267311	0.864437	-1.858693
C	-3.834030	-0.053271	-2.765028
C	-2.438674	-0.289583	-2.486888
C	-1.646727	-1.205598	-3.162880
C	2.764046	-1.083034	-1.179651
C	1.278477	2.348522	1.888116
C	-3.179098	2.091583	0.029880
C	0.324928	1.767191	-3.871948
S	0.069673	2.427604	-2.199750
H	1.187086	1.096544	-3.910236
H	-0.566199	1.201576	-4.171393
H	0.455158	2.597920	-4.571831
H	-2.112094	-1.771793	-3.963972
H	3.699469	-1.606602	-1.351601
H	1.748198	2.924314	2.679655
H	-4.130217	2.567704	0.246732
H	0.130953	-3.062721	-4.404649
H	2.604297	-2.992645	-3.294302
H	4.861836	-0.231933	0.558654
H	4.031957	1.705200	2.267745
H	-0.523626	4.125312	3.204640
H	-3.030423	3.957439	2.183538
H	-5.258336	1.285314	-1.746647
H	-4.396401	-0.544717	-3.548753
C	3.110610	-2.672206	3.876562
C	1.627883	-2.930259	4.169573
C	0.824964	-3.286235	2.910306
C	-0.653582	-3.652158	3.152949
C	-1.294747	-4.166068	1.854168
C	-1.463791	-2.472027	3.713754

H	3.234743	-1.839950	3.172523
H	3.585447	-3.555754	3.430883
H	1.537632	-3.746320	4.901972
H	1.198504	-2.040820	4.648702
H	0.866735	-2.449458	2.198131
H	1.317148	-4.137441	2.414841
H	-0.680653	-4.468806	3.892868
H	-2.337087	-4.467370	2.018010
H	-0.753032	-5.034304	1.457728
H	-1.286767	-3.381510	1.087508
H	-2.512902	-2.756218	3.864384
H	-1.440589	-1.627551	3.013733
H	-1.075701	-2.126808	4.678875
H	3.663893	-2.422951	4.790267

ω'

O	-0.331164	0.351672	1.338899
Fe	0.058227	1.036971	-0.079841
N	0.924047	-0.632223	-0.810392
N	1.858076	1.677569	0.561998
N	-0.744703	2.829498	0.391206
N	-1.661046	0.542865	-1.018980
C	0.304539	-1.659212	-1.471735
C	1.229233	-2.744340	-1.695191
C	2.417621	-2.362826	-1.152151
C	2.217382	-1.045523	-0.598610
C	3.026039	0.962697	0.580100
C	4.068963	1.720759	1.228636
C	3.513559	2.905105	1.603925
C	2.132200	2.863940	1.187575
C	-0.119311	3.861466	1.046338
C	-1.059121	4.921296	1.322481
C	-2.262669	4.512846	0.837403
C	-2.054752	3.208016	0.256796
C	-2.854865	1.232979	-0.975556
C	-3.886147	0.482456	-1.646760
C	-3.314664	-0.671638	-2.085940
C	-1.931889	-0.635668	-1.677658
C	-1.022116	-1.660390	-1.892053
C	3.200366	-0.306896	0.042880
C	1.219513	3.889319	1.406208
C	-3.046432	2.462965	-0.370165
C	0.764139	0.970436	-3.663196
S	0.328469	2.212739	-2.412347
H	1.678206	0.435166	-3.393864
H	-0.052213	0.241652	-3.741066
H	0.880168	1.460866	-4.634227
H	-1.378940	-2.542836	-2.414398
H	4.184395	-0.756654	0.133302
H	1.580298	4.776688	1.917244
H	-4.045089	2.887130	-0.403772
H	0.983987	-3.668929	-2.202061
H	3.351684	-2.909091	-1.119975
H	5.085952	1.376758	1.367445

H	3.978218	3.737507	2.116799
H	-0.812080	5.848037	1.824460
H	-3.210843	5.034815	0.855021
H	-4.911802	0.810879	-1.756243
H	-3.774724	-1.488471	-2.627352
C	-1.254558	-7.385816	2.692766
C	-0.757004	-5.994835	2.282957
C	-1.681089	-4.865094	2.759271
C	-1.282693	-3.447096	2.303760
C	0.072734	-2.997726	2.872202
C	-2.375354	-2.430755	2.669911
H	-2.250161	-7.589299	2.278609
H	-1.328705	-7.473650	3.783966
H	0.255057	-5.843933	2.680091
H	-0.664761	-5.948089	1.187792
H	-2.700631	-5.075249	2.401453
H	-1.738384	-4.884022	3.859179
H	-1.195856	-3.460449	1.205576
H	0.309189	-1.982145	2.536448
H	0.890567	-3.656290	2.557729
H	0.050464	-2.993518	3.970929
H	-2.110735	-1.429822	2.311885
H	-2.507006	-2.375100	3.759236
H	-3.342987	-2.709497	2.233179
H	-0.579455	-8.175033	2.340981

ω''

O	-0.749151	0.146840	1.042156
Fe	0.170632	0.709470	-0.170950
N	0.538721	-1.121761	-0.933506
N	1.882822	0.601854	0.886842
N	-0.037515	2.644856	0.367852
N	-1.349368	0.937694	-1.482061
C	-0.233437	-1.809911	-1.831664
C	0.276395	-3.147572	-2.012636
C	1.366170	-3.262937	-1.204542
C	1.519827	-1.995628	-0.531783
C	2.688258	-0.495539	1.037292
C	3.752381	-0.213630	1.970829
C	3.578014	1.071411	2.383293
C	2.404986	1.567770	1.704334
C	0.743278	3.336008	1.261099
C	0.198379	4.653170	1.487144
C	-0.927825	4.745651	0.729738
C	-1.064299	3.488484	0.033999
C	-2.202209	2.017725	-1.578943
C	-3.249079	1.739160	-2.529676
C	-3.033942	0.480203	-2.998881
C	-1.858226	-0.020383	-2.330148
C	-1.342539	-1.296895	-2.497364
C	2.524672	-1.709405	0.380789
C	1.886583	2.846385	1.874071
C	-2.083569	3.197649	-0.864872
C	1.602082	0.575316	-3.528268

S	1.361116	1.821615	-2.229627
H	2.178526	-0.277842	-3.161712
H	0.621359	0.212481	-3.860166
H	2.104367	1.037308	-4.383336
H	-1.857249	-1.955890	-3.189950
H	3.239770	-2.496308	0.599919
H	2.408983	3.508939	2.557334
H	-2.834724	3.962774	-1.034315
H	-0.154711	-3.887547	-2.674824
H	2.016560	-4.117065	-1.065632
H	4.522863	-0.917526	2.258427
H	4.174539	1.644179	3.081802
H	0.633430	5.394968	2.144656
H	-1.610301	5.580265	0.632530
H	-4.041033	2.429514	-2.790513
H	-3.614721	-0.078461	-3.721781
C	0.602109	-4.073926	3.303985
C	-0.641341	-4.291655	2.433699
C	-1.654337	-3.143899	2.551130
C	-2.884473	-3.253456	1.627716
C	-3.715243	-1.962346	1.691121
C	-3.758149	-4.477347	1.944790
H	0.332976	-3.985318	4.364100
H	1.124463	-3.152420	3.018714
H	-0.334782	-4.393801	1.382456
H	-1.111186	-5.244760	2.711515
H	-1.997805	-3.069316	3.595523
H	-1.142036	-2.198582	2.326815
H	-2.512888	-3.359461	0.596050
H	-4.571937	-2.006757	1.006453
H	-3.105963	-1.091468	1.425496
H	-4.109970	-1.802116	2.703824
H	-4.629043	-4.521921	1.278906
H	-4.133503	-4.430113	2.976206
H	-3.209364	-5.419320	1.833515
H	1.310490	-4.906398	3.210258

ω-1

O	0.500002	-0.467062	0.880665
Fe	0.448944	0.417800	-0.479018
N	0.975840	-1.124493	-1.667295
N	2.382271	0.972241	-0.357681
N	-0.095804	2.120463	0.461501
N	-1.496825	0.049587	-0.882334
C	0.140576	-2.050268	-2.234046
C	0.905428	-3.086049	-2.885541
C	2.218079	-2.779041	-2.697591
C	2.252964	-1.555800	-1.933044
C	3.468264	0.268362	-0.805511
C	4.690652	0.933633	-0.423008
C	4.327400	2.051035	0.263560
C	2.884581	2.061067	0.302698
C	0.742886	3.055528	1.015992
C	-0.020980	4.060599	1.715413

C	-1.330635	3.715410	1.587935
C	-1.366206	2.505379	0.802113
C	-2.588668	0.716796	-0.366733
C	-3.806884	0.059733	-0.769143
C	-3.447247	-1.016313	-1.521136
C	-2.006006	-1.026226	-1.574847
C	-1.249685	-2.001804	-2.207390
C	3.415678	-0.911189	-1.538062
C	2.127081	3.041096	0.933469
C	-2.532532	1.848343	0.428896
C	-0.013635	0.864191	-4.074503
S	0.021202	1.914218	-2.593315
H	0.915356	0.299361	-4.186565
H	-0.842505	0.150875	-3.986131
H	-0.190378	1.488333	-4.955453
H	-1.786251	-2.803999	-2.704836
H	4.360084	-1.363710	-1.824620
H	2.664066	3.850948	1.417748
H	-3.473797	2.265215	0.773099
H	0.477599	-3.929953	-3.411628
H	3.093150	-3.318341	-3.037245
H	5.685723	0.578997	-0.659559
H	4.961433	2.806088	0.710482
H	0.406684	4.910420	2.231892
H	-2.204313	4.223745	1.975308
H	-4.801466	0.392681	-0.501306
H	-4.085282	-1.751504	-1.994816
C	0.793726	-2.045041	5.469185
C	-0.250398	-1.569548	4.452371
C	-1.085844	-2.721767	3.875724
C	-2.075824	-2.317634	2.764992
C	-3.179568	-1.377498	3.274439
C	-2.695068	-3.562865	2.112074
H	1.498110	-2.751173	5.011642
H	0.320916	-2.554907	6.318695
H	-0.908050	-0.833359	4.933748
H	0.249558	-1.046103	3.626502
H	-0.397305	-3.476497	3.467265
H	-1.636480	-3.221553	4.689488
H	-1.501947	-1.781966	1.995745
H	-3.866988	-1.108844	2.462771
H	-2.769688	-0.446145	3.679818
H	-3.770632	-1.857749	4.066883
H	-3.365159	-3.286144	1.288567
H	-3.281957	-4.141239	2.839035
H	-1.921117	-4.225971	1.706413
H	1.376566	-1.205914	5.867396

ω-2R

O	0.524058	-0.135727	1.128489
Fe	0.532114	0.568473	-0.333799
N	1.473782	-0.976355	-1.225541
N	2.309886	1.471630	-0.043620
N	-0.416019	2.239139	0.291129

N	-1.245791	-0.186097	-0.927495	H	4.836959	-0.621179	-0.912167
C	0.893892	-2.093954	-1.764508	H	1.852665	4.534867	1.344119
C	1.905115	-3.042390	-2.164984	H	-3.780755	1.805206	0.153237
C	3.107530	-2.486344	-1.852531	H	1.703193	-4.002667	-2.622184
C	2.829116	-1.199014	-1.263212	H	4.099161	-2.894898	-1.999813
C	3.550167	0.928520	-0.248594	H	5.640868	1.646975	0.135718
C	4.577446	1.841437	0.192339	H	4.368517	3.847546	1.084612
C	3.939347	2.945468	0.667584	H	-0.642341	5.254281	1.715333
C	2.524561	2.700245	0.520566	H	-3.039418	4.083102	1.228569
C	0.167731	3.366289	0.814660	H	-4.581607	-0.405755	-1.014259
C	-0.844641	4.288963	1.269158	H	-3.312384	-2.534059	-2.109774
C	-2.047059	3.700911	1.026303	C	-1.341777	-4.734549	1.491359
C	-1.768345	2.424135	0.414038	C	-2.312172	-3.705561	2.083413
C	-2.495935	0.325455	-0.647098	C	-1.600420	-2.635742	2.923850
C	-3.517523	-0.580383	-1.109035	C	-2.507164	-1.513031	3.466942
C	-2.880045	-1.648914	-1.660882	C	-1.657837	-0.408380	4.114662
C	-1.464485	-1.404761	-1.529876	C	-3.568047	-2.030114	4.451322
C	-0.474203	-2.292593	-1.923929	H	-0.788566	-5.257737	2.281535
C	3.799630	-0.316147	-0.813747	H	-0.604923	-4.249477	0.839311
C	1.532543	3.592420	0.910469	H	-2.861963	-3.214377	1.267335
C	-2.743632	1.529469	-0.010020	H	-3.063757	-4.228714	2.689876
C	0.550496	0.524089	-3.985806	H	-1.088337	-3.121421	3.770102
S	0.184048	1.718654	-2.668148	H	-0.813838	-2.173630	2.312082
H	1.564815	0.127264	-3.895100	H	-3.030849	-1.067670	2.606166
H	-0.155220	-0.312924	-3.914643	H	-2.285419	0.419259	4.469268
H	0.413805	1.006304	-4.958271	H	-0.928262	-0.007357	3.402513
H	-0.793292	-3.227704	-2.373925	H	-1.105517	-0.798483	4.980550

H	-4.189766	-1.207852	4.827074
H	-3.093021	-2.507719	5.319189
H	-4.237362	-2.766161	3.991765
H	-1.869749	-5.492100	0.898970

ω-2S

O	0.517976	-0.125309	1.130312
Fe	0.530335	0.574104	-0.334177
N	1.469128	-0.976185	-1.219490
N	2.310145	1.473353	-0.044333
N	-0.414133	2.249519	0.283595
N	-1.248754	-0.177487	-0.928270
C	0.886972	-2.093993	-1.755613
C	1.896198	-3.046530	-2.151416
C	3.099663	-2.492708	-1.839066
C	2.823897	-1.202669	-1.254429
C	3.549243	0.926204	-0.245702
C	4.578369	1.837786	0.193706
C	3.942590	2.945118	0.664349
C	2.527348	2.703260	0.516066
C	0.171912	3.376813	0.804202
C	-0.838609	4.303906	1.253827
C	-2.042276	3.718421	1.010954
C	-1.766134	2.438750	0.403571
C	-2.497900	0.338407	-0.651442
C	-3.521284	-0.566372	-1.111487
C	-2.885922	-1.638620	-1.658532
C	-1.469892	-1.397690	-1.526616
C	-0.481433	-2.289486	-1.916297
C	3.796153	-0.320979	-0.806405
C	1.537204	3.599465	0.901447
C	-2.743230	1.545359	-0.018996
C	0.554316	0.517534	-3.986261
S	0.188642	1.717518	-2.673322
H	1.567412	0.118334	-3.892352
H	-0.153751	-0.317356	-3.913516
H	0.420644	0.996765	-4.960620
H	-0.802395	-3.225336	-2.363411
H	4.832790	-0.629151	-0.902294
H	1.859263	4.542494	1.332391
H	-3.779837	1.824563	0.141652
H	1.692322	-4.007791	-2.605668
H	4.090388	-2.904463	-1.983506
H	5.641338	1.640249	0.139267
H	4.373613	3.847415	1.078987
H	-0.634328	5.270191	1.696998
H	-3.033889	4.104105	1.210222
H	-4.585031	-0.388596	-1.018781
H	-3.320032	-2.524236	-2.104769
C	-1.300752	-4.713879	1.501959
C	-2.318328	-3.736280	2.110455
C	-1.595703	-2.639078	2.917281
C	-2.487872	-1.491434	3.411872
C	-1.687375	-0.387743	4.113417

H	-0.732973	-5.228236	2.288902
H	-0.581265	-4.189455	0.861951
H	-2.845491	-3.242761	1.278683
H	-1.091632	-3.104773	3.779445
H	-0.801649	-2.207531	2.292930
H	-3.021918	-1.059240	2.553031
H	-2.340203	0.425170	4.454542
H	-0.937991	0.036388	3.435436
H	-1.159949	-0.780669	4.992237
H	-1.797254	-5.483321	0.896814
C	-3.360303	-4.497479	2.945253
H	-2.885338	-4.990655	3.804292
H	-4.141588	-3.834711	3.332954
H	-3.852658	-5.275458	2.348318
H	-3.258709	-1.873816	4.094942

ω-3R

O	0.306165	-0.739987	0.824862
Fe	0.459233	0.218314	-0.476098
N	1.701870	-1.048423	-1.434528
N	2.052355	1.205743	0.263958
N	-0.763226	1.656786	0.243184
N	-1.103944	-0.566403	-1.488163
C	1.347313	-2.095501	-2.243033
C	2.512339	-2.862062	-2.613747
C	3.578409	-2.268879	-2.010223
C	3.063269	-1.140313	-1.273291
C	3.364384	0.820698	0.189446
C	4.196881	1.724105	0.947069
C	3.367984	2.661800	1.481478
C	2.031601	2.324087	1.053204
C	-0.403909	2.714629	1.041430
C	-1.575510	3.445465	1.461034
C	-2.648689	2.809618	0.917147
C	-2.130275	1.697306	0.157221
C	-2.433235	-0.223900	-1.351852
C	-3.257315	-1.114126	-2.130631
C	-2.421469	-2.005481	-2.729633
C	-1.082555	-1.669108	-2.312600
C	0.053588	-2.380721	-2.668321
C	3.842330	-0.272787	-0.522408
C	0.893509	3.038571	1.408605
C	-2.916289	0.815951	-0.575947
C	1.047909	0.841794	-4.026536
S	0.347233	1.740816	-2.612934
H	2.075621	0.525891	-3.829968
H	0.441872	-0.053068	-4.215315
H	1.006556	1.478137	-4.915480
H	-0.080066	-3.242343	-3.315360
H	4.910842	-0.462386	-0.488773
H	1.029736	3.909636	2.042161
H	-3.991013	0.966900	-0.553990
H	2.497506	-3.736733	-3.251500
H	4.621800	-2.554881	-2.049037

H	5.271634	1.637788	1.043706
H	3.618775	3.506476	2.110330
H	-1.559582	4.325964	2.090492
H	-3.698067	3.060793	1.003428
H	-4.335847	-1.051024	-2.198203
H	-2.672416	-2.828778	-3.386250
C	-3.383679	-3.386440	1.609861
C	-2.260687	-2.823544	2.488730
C	-2.681542	-1.561014	3.254354
C	-1.564281	-0.893818	4.081703
C	-1.058856	-1.793474	5.220450
C	-2.040515	0.456821	4.638535
H	-3.691682	-2.658134	0.849125
H	-4.270105	-3.639491	2.206267
H	-1.936379	-3.600180	3.194220
H	-1.390896	-2.583492	1.863278
H	-3.057160	-0.826169	2.526972
H	-3.530167	-1.797572	3.917199
H	-0.724617	-0.700606	3.398806
H	-0.272628	-1.290842	5.797276
H	-0.641169	-2.734386	4.846599
H	-1.873221	-2.040897	5.915754
H	-1.245155	0.955055	5.206997
H	-2.897060	0.325206	5.314252
H	-2.349973	1.133211	3.832601
H	-3.064497	-4.295809	1.086581

ω-3S

O	-0.288054	-0.873137	0.720681
Fe	-0.157459	-0.130303	-0.716388
N	0.598997	-1.729106	-1.692754
N	1.716579	0.529607	-0.367125
N	-0.888790	1.622147	-0.041345
N	-2.006255	-0.662303	-1.331119
C	-0.113995	-2.771332	-2.247795
C	0.786702	-3.795600	-2.713918
C	2.048463	-3.374168	-2.427989
C	1.924249	-2.093307	-1.776619
C	2.884994	-0.143231	-0.608249
C	4.008397	0.594698	-0.083665
C	3.502847	1.722042	0.487797
C	2.071640	1.671421	0.309785
C	-0.187426	2.619360	0.582575
C	-1.082913	3.659134	1.030259
C	-2.337486	3.275445	0.666908
C	-2.203829	2.000043	0.004528
C	-3.174400	0.023602	-1.107167
C	-4.302614	-0.741840	-1.580607
C	-3.803966	-1.904395	-2.081080
C	-2.370655	-1.843126	-1.923007
C	-1.493540	-2.836749	-2.341361
C	2.989493	-1.358474	-1.278078
C	1.191694	2.648025	0.752259
C	-3.271175	1.267182	-0.501496

C	1.711920	1.143826	-3.586182
S	-0.012215	0.731285	-3.192695
H	2.112424	1.890267	-2.895527
H	2.322599	0.235982	-3.505311
H	1.770361	1.506049	-4.616818
H	-1.922848	-3.724405	-2.795386
H	3.984376	-1.775190	-1.402468
H	1.613726	3.504084	1.269713
H	-4.263647	1.694016	-0.394239
H	0.475567	-4.714452	-3.194288
H	2.987499	-3.876962	-2.621453
H	5.040923	0.276521	-0.151244
H	4.033956	2.523309	0.985580
H	-0.774341	4.558120	1.548348
H	-3.275174	3.792309	0.826041
H	-5.333757	-0.416886	-1.525912
H	-4.339363	-2.733136	-2.526424
C	2.566196	-0.163160	4.293343
C	1.140966	-0.708663	4.146758
C	0.067332	0.341007	4.467307
C	-1.387054	-0.119627	4.242970
C	-2.356501	1.061243	4.406698
C	-1.788608	-1.274225	5.174279
H	2.752181	0.202365	5.311819
H	2.741500	0.671681	3.603200
H	0.989227	-1.067117	3.120210
H	1.022859	-1.579764	4.804965
H	0.182611	0.679421	5.510226
H	0.249790	1.224218	3.837285
H	-1.459644	-0.475202	3.205192
H	-3.393189	0.752883	4.221825
H	-2.118494	1.871943	3.707497
H	-2.310276	1.471455	5.425117
H	-2.829532	-1.573786	5.000333
H	-1.701212	-0.976538	6.228618
H	-1.162573	-2.160267	5.024159
H	3.314370	-0.934643	4.075108

RCs of conformer 2:

ω

O	-0.325336	-0.561999	0.723644
Fe	-0.091511	0.560815	-0.424836
N	0.680193	-0.750881	-1.748243
N	1.771265	1.015096	0.195697
N	-0.828550	2.077495	0.687131
N	-1.906586	0.344288	-1.284742
C	-0.013379	-1.525143	-2.640041
C	0.875716	-2.448034	-3.303249
C	2.118520	-2.227418	-2.793919
C	1.986883	-1.169665	-1.821482
C	2.930567	0.369819	-0.143780

C	4.042581	0.909375	0.601284
C	3.538153	1.891031	1.397471
C	2.119049	1.943598	1.139670
C	-0.129111	2.862236	1.570575
C	-1.027334	3.745225	2.274968
C	-2.279542	3.476379	1.816303
C	-2.143833	2.436565	0.824771
C	-3.085302	0.942489	-0.889888
C	-4.188530	0.416808	-1.654037
C	-3.674449	-0.511886	-2.505280
C	-2.254312	-0.564342	-2.259248
C	-1.378385	-1.432566	-2.893504
C	3.038231	-0.650319	-1.081075
C	1.241575	2.814816	1.774680
C	-3.202836	1.902516	0.100354
C	0.209309	1.685440	-3.887775
S	-0.074563	2.438516	-2.259881
H	1.144467	1.120109	-3.909733
H	-0.615363	0.997010	-4.110576
H	0.218154	2.468934	-4.651292
H	-1.795063	-2.108570	-3.633962
H	4.023936	-1.074052	-1.247203
H	1.661332	3.500773	2.504052
H	-4.197229	2.281474	0.314620
H	0.571476	-3.163984	-4.056122
H	3.047828	-2.724478	-3.041403
H	5.066945	0.570925	0.511888
H	4.060967	2.527175	2.100186
H	-0.721491	4.467759	3.020780
H	-3.217448	3.933682	2.104354
H	-5.218782	0.728426	-1.538784
H	-4.195577	-1.123348	-3.230899
C	1.483010	-4.803951	4.769199
C	1.549574	-3.614096	3.803297
C	0.490049	-3.636598	2.687368
C	-0.984795	-3.566504	3.137261
C	-1.911057	-3.714977	1.919547
C	-1.301666	-2.264885	3.890905
H	1.557794	-5.756084	4.227965
H	0.543526	-4.819650	5.333526
H	1.474202	-2.677714	4.371819
H	2.542041	-3.596018	3.331725
H	0.675951	-2.797453	2.002725
H	0.629312	-4.555093	2.096274
H	-1.184322	-4.411565	3.813839
H	-2.966549	-3.705349	2.219707
H	-1.722486	-4.656184	1.387427
H	-1.751821	-2.889138	1.215284
H	-2.360021	-2.228593	4.178527
H	-1.094859	-1.395554	3.254341
H	-0.709390	-2.163948	4.807903
H	2.302668	-4.770123	5.497035

ω-1

O	0.554468	0.003896	0.888818	S	-0.750426	1.106705	-2.988721
Fe	0.188189	0.415026	-0.638129	H	0.359278	-0.693866	-4.201533
N	0.955720	-1.273886	-1.429361	H	-1.293556	-1.123170	-3.715737
N	1.944612	1.350128	-0.956103	H	-1.051451	-0.028235	-5.092808
N	-0.654031	2.177222	-0.122320	H	-1.422547	-3.692936	-1.565093
N	-1.646385	-0.431452	-0.637275	H	4.278915	-0.877895	-2.008352
C	0.311502	-2.470552	-1.599885	H	1.701168	4.622063	-0.088045
C	1.237102	-3.480230	-2.053710	H	-3.933454	1.736602	0.638595
C	2.454989	-2.879732	-2.147540	H	0.972553	-4.508213	-2.266380
C	2.271150	-1.504141	-1.752709	H	3.399222	-3.311824	-2.453587
C	3.122736	0.773493	-1.349531	H	5.213026	1.543793	-1.618255
C	4.186091	1.749383	-1.344368	H	4.110501	3.888773	-0.813784
C	3.633005	2.925703	-0.941586	H	-0.661783	5.407164	0.736495
C	2.235160	2.662436	-0.696551	H	-3.054236	4.173499	1.067145
C	-0.010616	3.380629	0.028476	H	-4.894900	-0.651838	0.125440
C	-0.923636	4.375872	0.537234	H	-3.833441	-2.953464	-0.831859
C	-2.123359	3.756734	0.704458	C	2.178780	-1.600262	3.826435
C	-1.944740	2.386622	0.287621	C	1.264575	-2.492430	4.676319
C	-2.814470	0.128233	-0.162666	C	-0.112666	-1.876915	4.982704
C	-3.877312	-0.845044	-0.189146	C	-1.055197	-1.671986	3.777912
C	-3.344553	-2.000931	-0.671781	C	-2.300768	-0.880651	4.206739
C	-1.950092	-1.741423	-0.933574	C	-1.466570	-3.002942	3.129102
C	-1.045376	-2.690843	-1.385103	H	2.354867	-0.637274	4.323254
C	3.282882	-0.556107	-1.720015	H	1.750794	-1.384509	2.841844
C	1.327231	3.615864	-0.250711	H	1.137773	-3.465387	4.182603
C	-2.954226	1.431546	0.283130	H	1.763862	-2.706715	5.631829
C	-0.662953	-0.317510	-4.111869	H	-0.628904	-2.508842	5.722426

H	0.045819	-0.904407	5.472853
H	-0.531813	-1.077016	3.017923
H	-2.964195	-0.696300	3.352885
H	-2.027064	0.091864	4.634162
H	-2.876739	-1.428233	4.965826
H	-2.148744	-2.831055	2.287830
H	-1.982258	-3.650534	3.852304
H	-0.602951	-3.555350	2.742390
H	3.155153	-2.075606	3.671119

ω-2R

O	0.695119	-0.155251	0.851920
Fe	0.394194	0.321866	-0.669885
N	0.578093	-1.534682	-1.435999
N	2.332656	0.676868	-1.090821
N	0.147451	2.265721	-0.179035
N	-1.607899	0.069158	-0.565838
C	-0.403415	-2.484521	-1.537735
C	0.152450	-3.735792	-1.993220
C	1.488276	-3.532810	-2.157923
C	1.745971	-2.157878	-1.804312
C	3.261726	-0.235882	-1.514053
C	4.568090	0.373402	-1.585802
C	4.415094	1.669216	-1.199151
C	3.016892	1.844851	-0.887089
C	1.129688	3.221628	-0.094904
C	0.583678	4.456370	0.415042
C	-0.737261	4.232098	0.650414
C	-0.999259	2.863658	0.274143
C	-2.529452	0.965223	-0.065005
C	-3.836110	0.358494	-0.017600
C	-3.699693	-0.913485	-0.481129
C	-2.306009	-1.093380	-0.805777
C	-1.751699	-2.280511	-1.260556
C	2.996114	-1.558879	-1.845614
C	2.460882	3.036381	-0.436027
C	-2.248741	2.258512	0.341685
C	-0.807765	-0.188629	-4.082111
S	-0.406897	1.217353	-3.004982
H	0.046580	-0.860400	-4.197920
H	-1.634361	-0.755340	-3.635749
H	-1.135151	0.185343	-5.056724
H	-2.420928	-3.125487	-1.391017
H	3.833847	-2.171971	-2.163610
H	3.127638	3.885692	-0.322671
H	-3.072728	2.853012	0.723736
H	-0.418851	-4.640382	-2.158446
H	2.242740	-4.236074	-2.486801
H	5.470686	-0.137705	-1.895604
H	5.165825	2.445424	-1.123352
H	1.152708	5.364437	0.568213
H	-1.481211	4.918070	1.034801
H	-4.731864	0.856408	0.330939
H	-4.459352	-1.677304	-0.588165

C	-0.053570	0.763814	4.428152
C	-1.477024	0.226626	4.230926
C	-1.537584	-1.172469	3.592182
C	-0.911036	-2.326207	4.402315
C	-0.927579	-3.621684	3.576018
C	-1.604459	-2.541667	5.756384
H	0.494196	0.758946	3.478421
H	0.515127	0.160815	5.146049
H	-2.013375	0.232684	5.190127
H	-2.024416	0.918017	3.575632
H	-2.590586	-1.425095	3.390480
H	-1.036920	-1.121011	2.615976
H	0.141801	-2.075766	4.597232
H	-0.448873	-4.447743	4.116700
H	-0.400531	-3.489837	2.623613
H	-1.958067	-3.927531	3.348564
H	-1.150831	-3.377673	6.303156
H	-2.668826	-2.775666	5.616683
H	-1.540992	-1.655421	6.397581
H	-0.070424	1.793038	4.807200

ω-2S

O	0.320009	-0.156700	1.056001
Fe	0.319252	0.680143	-0.334800
N	1.622915	-0.560033	-1.246141
N	1.850179	1.868440	0.216776
N	-0.992125	2.069931	0.320109
N	-1.214388	-0.332490	-1.175179
C	1.325732	-1.707184	-1.932935
C	2.536116	-2.400364	-2.302440
C	3.572367	-1.660578	-1.821204
C	2.993610	-0.516239	-1.159881
C	3.186673	1.593878	0.099355
C	3.969799	2.634040	0.722084
C	3.086276	3.542207	1.218644
C	1.766698	3.050562	0.902092
C	-0.691814	3.226729	0.996083
C	-1.903047	3.891039	1.413584
C	-2.938954	3.116025	0.993026
C	-2.359799	1.984065	0.310348
C	-2.561075	-0.095398	-0.991435
C	-3.338058	-1.126915	-1.631544
C	-2.455430	-1.996729	-2.194286
C	-1.133492	-1.504863	-1.893034
C	0.043749	-2.144927	-2.250687
C	3.726593	0.483972	-0.538903
C	0.587190	3.694563	1.257190
C	-3.097391	0.970474	-0.289655
C	0.670742	1.008845	-3.954236
S	-0.041221	1.974223	-2.590929
H	1.731797	0.805681	-3.788606
H	0.142718	0.049790	-4.025505
H	0.526878	1.550503	-4.893819
H	-0.042651	-3.073735	-2.806317

H	4.808387	0.392861	-0.552799
H	0.674722	4.630545	1.800324
H	-4.178926	1.028692	-0.217489
H	2.569601	-3.330119	-2.855980
H	4.634392	-1.856177	-1.897249
H	5.051454	2.651236	0.762124
H	3.289816	4.461236	1.753233
H	-1.936177	4.826028	1.958127
H	-4.001337	3.282356	1.117284
H	-4.419822	-1.165725	-1.638296
H	-2.662593	-2.899081	-2.755321
C	-2.874102	-5.130905	2.557636
C	-2.229183	-4.012115	1.729704
C	-1.960397	-2.713499	2.510183
C	-0.956473	-2.805502	3.678910
C	-0.889498	-1.458539	4.416508
C	0.445556	-3.224463	3.208394
H	-3.814185	-4.792993	3.012767
H	-2.218735	-5.467359	3.369220
H	-1.295298	-4.379905	1.284152
H	-2.886842	-3.770525	0.883119
H	-1.595311	-1.948345	1.811546
H	-2.919173	-2.341563	2.903866
H	-1.318442	-3.560598	4.393475
H	-0.206610	-1.508035	5.273932
H	-1.876673	-1.159158	4.791059
H	-0.529818	-0.670662	3.743061
H	1.147417	-3.253851	4.051312
H	0.831111	-2.507611	2.472698
H	0.446150	-4.218910	2.746840
H	-3.101541	-6.005087	1.935538

ω-3R

O	0.845069	0.145269	0.927039
Fe	0.654179	0.456482	-0.654027
N	1.254902	-1.380674	-1.230535
N	2.536915	1.135798	-0.891325
N	0.010921	2.349402	-0.363848
N	-1.269154	-0.155060	-0.745672
C	0.476515	-2.500856	-1.352557
C	1.295662	-3.654627	-1.635699
C	2.584366	-3.218363	-1.675102
C	2.550230	-1.799061	-1.417162
C	3.656592	0.385353	-1.132869
C	4.834230	1.219632	-1.114308
C	4.409665	2.486690	-0.856958
C	2.974873	2.420556	-0.714177
C	0.791968	3.473263	-0.253944
C	-0.021155	4.616558	0.084619
C	-1.301241	4.168425	0.190158
C	-1.270211	2.753687	-0.093661
C	-2.388501	0.586692	-0.429801
C	-3.563498	-0.247223	-0.469507
C	-3.148927	-1.501014	-0.799347

C	-1.716189	-1.440506	-0.954522
C	-0.909963	-2.532394	-1.239859
C	3.670540	-0.982541	-1.376868
C	2.166908	3.515184	-0.429224
C	-2.392266	1.933892	-0.111499
C	-0.013932	-0.494931	-4.116448
S	-0.000352	1.030632	-3.131352
H	0.954459	-1.000688	-4.084113
H	-0.772003	-1.175430	-3.709143
H	-0.286293	-0.255971	-5.148717
H	-1.398852	-3.493628	-1.365999
H	4.635511	-1.449186	-1.549761
H	2.654930	4.478399	-0.316018
H	-3.348684	2.389493	0.125268
H	0.917480	-4.658489	-1.781427
H	3.485324	-3.789387	-1.859968
H	5.843575	0.865398	-1.280571
H	4.997136	3.391406	-0.766270
H	0.356325	5.621907	0.221151
H	-2.196010	4.728940	0.428916
H	-4.569066	0.098683	-0.267052
H	-3.743120	-2.397867	-0.919936
C	-0.346963	-0.849658	5.020925
C	-1.281613	-0.668778	3.818277
C	-2.112197	-1.915812	3.467376
C	-3.106550	-2.412085	4.538147
C	-3.759283	-3.728916	4.089477
C	-4.181582	-1.365596	4.869421
H	0.331920	-1.698145	4.866316
H	-0.897659	-1.031484	5.951972
H	-1.947919	0.185924	3.999792
H	-0.680408	-0.402243	2.940331
H	-2.671813	-1.716981	2.540632
H	-1.419097	-2.737285	3.230835
H	-2.544717	-2.620807	5.460531
H	-4.442631	-4.118895	4.853978
H	-3.005271	-4.499627	3.887970
H	-4.339896	-3.583815	3.168349
H	-4.886551	-1.748178	5.617812
H	-4.759299	-1.103303	3.972559
H	-3.747209	-0.441651	5.266278
H	0.270803	0.043036	5.172568

ω-3S

O	1.110970	-0.212457	0.745174
Fe	0.544054	0.682016	-0.484247
N	1.707699	-0.221529	-1.861774
N	1.938686	2.120234	-0.263746
N	-0.729397	1.743714	0.669921
N	-0.970192	-0.569048	-0.958084
C	1.422169	-1.365781	-2.558026
C	2.565598	-1.781418	-3.333961
C	3.552555	-0.876201	-3.090656
C	3.010140	0.091319	-2.167774

C	3.203374	2.125486	-0.788911	H	4.966837	3.496159	-0.573201
C	3.944182	3.265124	-0.303556	H	3.300290	4.859143	1.078037
C	3.108125	3.949111	0.524166	H	-1.599145	4.160507	2.808596
C	1.861622	3.221711	0.545306	H	-3.520247	2.285323	2.424710
C	-0.444935	2.897629	1.357652	H	-3.968425	-1.999928	-0.574943
C	-1.569778	3.282653	2.175859	H	-2.355705	-3.284410	-2.332351
C	-2.532978	2.341364	1.984326	C	0.587854	-4.309475	2.732179
C	-1.999218	1.387520	1.041742	C	-0.389090	-3.200785	2.320764
C	-2.218104	-0.616008	-0.372239	C	-1.240960	-2.646221	3.475894
C	-2.959527	-1.745872	-0.873798	C	-2.203051	-3.635228	4.166872
C	-2.150011	-2.390227	-1.757784	C	-3.256494	-4.199521	3.201207
C	-0.906030	-1.661172	-1.794194	C	-2.882468	-2.964670	5.371060
C	0.201214	-2.033024	-2.542030	H	0.070066	-5.206524	3.093344
C	3.709890	1.182696	-1.675105	H	1.255303	-3.968110	3.533903
C	0.751256	3.596017	1.293192	H	0.177314	-2.366995	1.888057
C	-2.695116	0.281475	0.567881	H	-1.041913	-3.567797	1.516919
C	-0.285227	1.264257	-3.993839	H	-0.564378	-2.230838	4.237740
S	-0.699328	1.999633	-2.385875	H	-1.828866	-1.795286	3.099653
H	0.793771	1.264895	-4.168229	H	-1.612433	-4.480267	4.550576
H	-0.636745	0.225124	-4.011579	H	-3.941034	-4.882314	3.719506
H	-0.801052	1.814600	-4.786251	H	-2.800552	-4.752973	2.373165
H	0.114539	-2.928756	-3.149565	H	-3.860032	-3.390237	2.767580
H	4.735171	1.310881	-2.008568	H	-3.543160	-3.663278	5.899238
H	0.834370	4.498873	1.890439	H	-3.492059	-2.109045	5.050340
H	-3.697922	0.119573	0.950637	H	-2.142791	-2.592284	6.090094
H	2.592067	-2.654005	-3.974187	H	1.216412	-4.611067	1.886018
H	4.558674	-0.850473	-3.489447				

TSs of conformer 1:

ω

O	-0.710490	0.157072	1.108997
Fe	0.223691	0.749752	-0.235103
N	0.554427	-1.100552	-0.933887
N	1.886520	0.628738	0.880457
N	-0.018936	2.688499	0.294564
N	-1.416484	0.918032	-1.441253
C	-0.264814	-1.821029	-1.767838
C	0.262957	-3.151144	-1.955028
C	1.404501	-3.228743	-1.216952
C	1.570770	-1.948582	-0.572109
C	2.728447	-0.443442	1.000052
C	3.789174	-0.147279	1.937615
C	3.576256	1.121090	2.377567
C	2.388227	1.599982	1.704995
C	0.748369	3.378442	1.196156
C	0.226918	4.712008	1.376143
C	-0.870873	4.815535	0.577416
C	-1.021346	3.545258	-0.089884
C	-2.230811	2.014804	-1.599180
C	-3.313689	1.703005	-2.501202
C	-3.145488	0.407569	-2.885142
C	-1.956348	-0.072570	-2.219495
C	-1.427863	-1.351207	-2.366748
C	2.595150	-1.644226	0.315774
C	1.865478	2.877398	1.855591
C	-2.051045	3.239759	-0.972123
C	1.413579	0.589568	-3.512341
S	1.348395	1.773337	-2.132717
H	1.946748	-0.321218	-3.228653
H	0.395414	0.318320	-3.813036
H	1.909181	1.071716	-4.360031
H	-1.966330	-2.040230	-3.010735
H	3.335708	-2.415001	0.506628
H	2.375833	3.545561	2.542750
H	-2.775369	4.020744	-1.182586
H	-0.194944	-3.914541	-2.571346
H	2.076230	-4.069646	-1.099543
H	4.582913	-0.832285	2.207477
H	4.159398	1.698229	3.083966
H	0.655840	5.457854	2.033291
H	-1.530191	5.663716	0.443314
H	-4.092163	2.396705	-2.792598
H	-3.756750	-0.181196	-3.557514
C	-3.419916	-4.178560	3.272676
C	-3.639438	-2.694088	3.586184
C	-3.197236	-1.776403	2.438418
C	-3.482444	-0.273794	2.652071
C	-3.208979	0.513332	1.387992
C	-2.716310	0.319435	3.847086
H	-2.361038	-4.387405	3.076152
H	-3.986211	-4.484177	2.383889

H	-4.703749	-2.520113	3.803806
H	-3.094021	-2.437819	4.503589
H	-2.122547	-1.904015	2.250839
H	-3.708185	-2.097518	1.518134
H	-4.564908	-0.172119	2.863537
H	-3.297325	1.598975	1.474519
H	-3.667779	0.124034	0.476157
H	-1.849846	0.367742	1.129809
H	-2.949507	1.384440	3.967411
H	-1.636061	0.228748	3.687541
H	-2.973262	-0.183251	4.785511
H	-3.737135	-4.816448	4.106071

ω'

O	74.691417	72.982393	36.745516
Fe	74.625507	74.294466	35.603566
N	75.654730	73.259681	34.229106
N	76.341915	75.016624	36.349507
N	73.557251	75.485304	36.845397
N	72.869136	73.641862	34.789359
C	75.139627	72.376210	33.312810
C	76.208240	71.747640	32.573850
C	77.374293	72.254333	33.061074
C	77.019188	73.186799	34.103575
C	77.613592	74.717311	35.942470
C	78.580654	75.410491	36.764750
C	77.872267	76.135173	37.670681
C	76.473212	75.887514	37.397898
C	74.072204	76.296137	37.822631
C	73.004203	76.931676	38.556295
C	71.836877	76.487625	38.014110
C	72.190999	75.579636	36.950257
C	71.591962	73.987377	35.164483
C	70.628584	73.261141	34.372088
C	71.336466	72.473286	33.516500
C	72.734757	72.720599	33.783609
C	73.789463	72.122427	33.101010
C	77.937151	73.875080	34.886973
C	75.424808	76.484550	38.085138
C	71.271331	74.888939	36.169638
C	74.511722	75.600407	32.370697
S	74.284185	76.163163	34.085405
H	75.515256	75.196122	32.216331
H	73.781236	74.816709	32.140809
H	74.335728	76.447017	31.700723
H	73.536421	71.400320	32.330313
H	78.990455	73.722524	34.671991
H	75.682442	77.162509	38.893291
H	70.218220	75.064712	36.366986
H	76.066899	71.014022	31.790178
H	78.388425	72.020753	32.762588
H	79.654468	75.339706	36.646482
H	78.241739	76.786704	38.452440
H	73.146770	77.623213	39.377004

H	70.822647	76.738498	38.297832
H	69.554402	73.354092	34.470303
H	70.963322	71.786983	32.766748
C	71.767905	67.370092	40.042433
C	72.833746	68.416869	39.698133
C	72.266228	69.596965	38.894451
C	73.319290	70.641260	38.437056
C	73.995858	71.356820	39.617147
C	72.676968	71.637133	37.495150
H	71.313479	66.952581	39.135268
H	70.961428	67.808429	40.643469
H	73.293553	68.781072	40.625447
H	73.640369	67.942443	39.121048
H	71.755452	69.200465	38.004945
H	71.492986	70.106487	39.489078
H	74.093939	70.097459	37.874883
H	74.710898	72.098746	39.248495
H	74.534212	70.656561	40.264668
H	73.250469	71.878832	40.232636
H	73.712656	72.431075	37.010233
H	72.023813	72.378678	37.963642
H	72.248710	71.219971	36.580792
H	72.194675	66.538132	40.614807

ω''

O	-0.655125	0.468163	1.006582
Fe	0.023130	1.290697	-0.368164
N	0.632271	-0.429418	-1.216911
N	1.768767	1.439419	0.606906
N	-0.522077	3.116037	0.291945
N	-1.722930	1.182512	-1.428879
C	-0.097616	-1.247003	-2.043898
C	0.649891	-2.443558	-2.345729
C	1.833789	-2.345446	-1.679470
C	1.807803	-1.090783	-0.967700
C	2.794269	0.532293	0.606700
C	3.864687	0.984249	1.468714
C	3.473191	2.180681	1.980594
C	2.164152	2.458813	1.430462
C	0.185679	3.910631	1.155887
C	-0.566533	5.102308	1.465725
C	-1.744552	5.010105	0.789693
C	-1.712333	3.763883	0.063707
C	-2.748923	2.095523	-1.429625
C	-3.829861	1.628097	-2.265163
C	-3.442834	0.424983	-2.772258
C	-2.124831	0.156579	-2.245213
C	-1.372417	-0.978005	-2.531107
C	2.822706	-0.643895	-0.129389
C	1.435990	3.612819	1.685484
C	-2.750994	3.294891	-0.730326
C	0.982240	1.499228	-3.727732
S	0.754142	2.587950	-2.288651
H	1.710104	0.711057	-3.520675

H	0.026114	1.032647	-3.990269
H	1.313568	2.109585	-4.573059
H	-1.819010	-1.719274	-3.187227
H	3.702192	-1.273377	-0.033104
H	1.876438	4.342174	2.358531
H	-3.634215	3.920775	-0.814398
H	0.298179	-3.246289	-2.981527
H	2.653932	-3.051733	-1.653720
H	4.787167	0.444375	1.640973
H	4.006160	2.831473	2.662148
H	-0.225364	5.892602	2.122302
H	-2.570872	5.709424	0.775487
H	-4.754612	2.164992	-2.434837
H	-3.984595	-0.229672	-3.443283
C	-0.663824	-4.212069	3.404582
C	-1.667344	-3.797317	2.322336
C	-2.015681	-2.303598	2.382000
C	-2.956912	-1.807113	1.265284
C	-3.060551	-0.296108	1.268477
C	-4.370238	-2.425986	1.361293
H	-1.062821	-4.020884	4.408669
H	0.272745	-3.649041	3.309727
H	-1.249863	-4.029205	1.331714
H	-2.575739	-4.406339	2.423204
H	-2.469451	-2.072939	3.358854
H	-1.087473	-1.721222	2.326052
H	-2.528371	-2.112609	0.298359
H	-3.657946	0.139647	0.465502
H	-1.776814	0.192003	1.020207
H	-3.268554	0.159598	2.241244
H	-5.016515	-2.059187	0.555373
H	-4.844783	-2.167151	2.316378
H	-4.334265	-3.519129	1.288265
H	-0.421769	-5.279563	3.340358

ω-1

O	74.411892	73.594797	37.283922
Fe	74.458956	74.591581	35.866095
N	75.066414	73.067673	34.712225
N	76.368022	75.045585	36.282286
N	73.830508	76.248716	36.846159
N	72.517286	74.205113	35.354572
C	74.267659	72.151090	34.072953
C	75.080910	71.142358	33.437089
C	76.378201	71.455646	33.706469
C	76.358603	72.651994	34.513471
C	77.486737	74.373580	35.870484
C	78.673267	74.987235	36.425364
C	78.252493	76.039826	37.175394
C	76.809551	76.071751	37.074392
C	74.620753	77.112329	37.557930
C	73.809345	78.130218	38.181434
C	72.517926	77.866996	37.839472
C	72.539288	76.686590	37.009991

C	71.401659	74.924767	35.710237
C	70.216368	74.301557	35.169924
C	70.629389	73.200551	34.483017
C	72.068716	73.152211	34.601949
C	72.879638	72.184715	34.016281
C	77.491287	73.263834	35.036382
C	76.004629	77.032608	37.671121
C	71.406572	76.079606	36.480747
C	74.217040	75.147469	32.424247
S	74.340309	76.107172	33.964938
H	75.074567	74.481763	32.299555
H	73.303220	74.543161	32.437632
H	74.156034	75.848187	31.586153
H	72.386954	71.394929	33.456946
H	78.453680	72.825084	34.790983
H	76.497675	77.793218	38.268996
H	70.448084	76.544310	36.691294
H	74.693054	70.309052	32.864798
H	77.275835	70.931444	33.403776
H	79.684765	74.643274	36.250224
H	78.845942	76.743990	37.744749
H	74.193951	78.934032	38.796347
H	71.622664	78.408920	38.116824
H	69.208390	74.673098	35.304763
H	70.030409	72.481552	33.938366
C	75.608784	72.252276	41.347068
C	74.550996	72.981789	40.511548
C	73.641467	72.010931	39.744426
C	72.625631	72.639815	38.794475
C	71.760819	73.737971	39.385107
C	71.857054	71.613292	37.981755
H	76.261803	71.641992	40.710941
H	75.146209	71.583591	42.084655
H	73.947369	73.617546	41.172732
H	75.040830	73.646618	39.790586
H	74.268896	71.317634	39.167591
H	73.088339	71.385287	40.469679
H	73.436062	73.243580	37.894298
H	71.076282	74.149654	38.636972
H	72.356399	74.562587	39.788601
H	71.148789	73.337262	40.210875
H	71.194282	72.086611	37.250278
H	71.230285	70.993826	38.644866
H	72.534885	70.938586	37.446732
H	76.243572	72.960272	41.892665

ω-2R

O	74.517745	73.725282	37.247082
Fe	74.499549	74.616598	35.754816
N	75.446449	73.153443	34.755254
N	76.263397	75.422781	36.262841
N	73.524909	76.204550	36.541852
N	72.698565	73.856476	35.148281
C	74.870014	72.089686	34.104460

C	75.891487	71.204717	33.599048	H	70.161720	75.863071	36.184862
C	77.089974	71.738852	33.963538	H	75.699173	70.294305	33.045485
C	76.801602	72.949731	34.693248	H	78.084838	71.357387	33.771548
C	77.508038	74.916196	36.003089	H	79.590945	75.578590	36.524086
C	78.525867	75.762270	36.587126	H	78.295154	77.628735	37.735799
C	77.876244	76.789758	37.194713	H	73.272164	79.076622	38.225242
C	76.461837	76.571202	36.981162	H	70.887913	78.033292	37.467654
C	74.098959	77.257503	37.205792	H	69.370853	73.716449	34.872570
C	73.079351	78.164592	37.674708	H	70.660747	71.651670	33.681624
C	71.881694	77.640370	37.294274	C	73.509036	70.333332	37.425296
C	72.167840	76.413032	36.592191	C	72.358629	71.233028	37.893577
C	71.451758	74.379799	35.390332	C	72.828472	72.458017	38.652013
C	70.435526	73.526983	34.819948	C	71.801105	73.432431	39.210178
C	71.083517	72.489940	34.221009	C	72.487068	74.627447	39.891195
C	72.497083	72.708094	34.428092	C	70.839576	72.737778	40.202009
C	73.505144	71.880138	33.944369	H	74.082903	69.957716	38.281549
C	77.767106	73.768809	35.266619	H	74.198522	70.883472	36.778375
C	75.462521	77.430676	37.416892	H	71.740898	71.536263	37.037741
C	71.199451	75.566331	36.066512	H	71.693685	70.642602	38.546592
C	74.463616	74.911893	32.262660	H	73.614814	72.222672	39.381738
S	74.275566	75.961107	33.736683	H	73.608836	73.177219	37.772357
H	75.430512	74.402984	32.254106	H	71.195087	73.810061	38.374192
H	73.669482	74.157249	32.242914	H	71.748479	75.363950	40.229345
H	74.360760	75.544848	31.376045	H	73.184650	75.123521	39.210869
H	73.200760	70.993777	33.395779	H	73.054322	74.295357	40.770954
H	78.805957	73.481475	35.135638	H	70.130418	73.465423	40.615619
H	75.770731	78.314797	37.966728	H	71.393713	72.298201	41.041342

H	70.260196	71.940470	39.724792
H	73.131972	69.466549	36.869952

ω-2S

O	74.520556	73.720810	37.241241
Fe	74.503365	74.612661	35.749391
N	75.448649	73.148697	34.749657
N	76.268101	75.416690	36.257588
N	73.530297	76.201572	36.536884
N	72.701548	73.854946	35.142545
C	74.871020	72.085173	34.099539
C	75.891547	71.199838	33.592866
C	77.090686	71.733534	33.955745
C	76.803737	72.944423	34.686023
C	77.512211	74.909497	35.996777
C	78.530876	75.753949	36.581644
C	77.882236	76.781050	37.190971
C	76.467617	76.564001	36.977450
C	74.105539	77.253361	37.201589
C	73.087083	78.162756	37.668680
C	71.888956	77.641335	37.285840
C	72.173682	76.413273	36.584392
C	71.455385	74.380891	35.382338
C	70.438258	73.528870	34.812338
C	71.085013	72.489502	34.216193
C	72.498771	72.705708	34.423970
C	73.505903	71.876368	33.940748
C	77.770124	73.762661	35.258919
C	75.469236	77.424202	37.413826
C	71.204415	75.568604	36.056911
C	74.465705	74.908914	32.257920
S	74.281941	75.958565	33.732254
H	75.431675	74.398260	32.247872
H	73.670209	74.155698	32.239320
H	74.362724	75.542062	31.371459
H	73.200530	70.989898	33.392886
H	78.808718	73.474853	35.126956
H	75.778442	78.307546	37.964360
H	70.167118	75.867774	36.173021
H	75.698187	70.289507	33.039537
H	78.085158	71.351677	33.762508
H	79.595790	75.569535	36.518003
H	78.301987	77.618928	37.733110
H	73.280902	79.074369	38.219553
H	70.895745	78.036494	37.457366
H	69.373862	73.720276	34.863537
H	70.661316	71.650982	33.677931
C	73.547312	70.389976	37.439903
C	72.352143	71.224424	37.927555
C	72.824696	72.474592	38.656811
C	71.802993	73.465042	39.176381
C	72.428844	74.654964	39.914056
H	74.124014	70.009131	38.293308
H	74.221784	70.987572	36.820653

H	71.762475	71.534807	37.053176
H	73.606198	72.252407	39.395786
H	73.610962	73.175608	37.767770
H	71.176705	73.821880	38.347806
H	71.659185	75.353520	40.261741
H	73.118523	75.199899	39.262844
H	72.994639	74.316006	40.790787
H	73.210576	69.525930	36.854265
C	71.432951	70.359534	38.820608
H	71.952810	70.054060	39.737810
H	70.520731	70.891606	39.110168
H	71.133817	69.448457	38.287729
H	71.117785	72.942566	39.865437

ω-3R

O	74.232911	73.372567	37.121514
Fe	74.345357	74.378656	35.711364
N	75.493128	73.042915	34.745717
N	75.981228	75.230227	36.497545
N	73.185497	75.840923	36.489687
N	72.680873	73.580153	34.824374
C	75.063843	71.995980	33.967512
C	76.188524	71.193097	33.551409
C	77.299651	71.760187	34.097243
C	76.854148	72.908107	34.849699
C	77.276888	74.811165	36.360262
C	78.163183	75.662962	37.123071
C	77.382974	76.604540	37.716162
C	76.020554	76.327790	37.314976
C	73.607788	76.860059	37.303314
C	72.483328	77.663018	37.718981
C	71.374774	77.109536	37.153493
C	71.821434	75.968577	36.391404
C	71.385343	74.024245	34.935609
C	70.503603	73.183938	34.159424
C	71.281939	72.231410	33.575645
C	72.639804	72.488483	33.997338
C	73.745686	71.738946	33.608229
C	77.691958	73.733747	35.589885
C	74.923498	77.091514	37.689667
C	70.978826	75.126949	35.674622
C	74.746272	74.935786	32.278157
S	74.285718	75.856570	33.777797
H	75.739347	74.489906	32.372376
H	74.018413	74.136677	32.098277
H	74.718970	75.625641	31.429325
H	73.565163	70.882607	32.965173
H	78.754077	73.508770	35.574342
H	75.107156	77.942135	38.339077
H	69.918382	75.360084	35.682596
H	76.118646	70.311387	32.926876
H	78.330473	71.439527	34.015202
H	79.237438	75.541192	37.180151
H	77.681303	77.420084	38.362723

H	72.550349	78.531714	38.361551
H	70.344285	77.431693	37.233787
H	69.432992	73.323092	34.077605
H	70.981777	71.424807	32.918661
C	71.805036	70.938684	37.143153
C	72.468250	71.875299	38.125971
C	71.555878	72.765038	38.944857
C	72.254220	73.692198	39.962095
C	72.946591	72.907441	41.087263
C	71.247181	74.693308	40.547983
H	71.088692	71.460970	36.500144
H	71.250438	70.151073	37.679691
H	73.249548	71.382895	38.713364
H	73.295760	72.719657	37.431070
H	70.947622	73.372618	38.259256
H	70.835822	72.123348	39.488274
H	73.022545	74.256454	39.417103
H	73.432862	73.589794	41.794842
H	73.717942	72.230464	40.704941
H	72.219979	72.306721	41.651762
H	71.731081	75.366896	41.265690
H	70.436113	74.173887	41.076706
H	70.796729	75.310818	39.762137
H	72.540608	70.441077	36.502076

ω-3S

O	75.097730	74.202547	37.415069
Fe	74.951930	74.844890	35.809152
N	75.235723	72.996785	35.086271
N	76.923535	75.208152	35.681490
N	74.646494	76.767530	36.445864
N	72.948133	74.586924	35.737108
C	74.270050	72.061722	34.826487
C	74.875683	70.832026	34.363033
C	76.218121	71.042636	34.340642
C	76.431907	72.400839	34.790712
C	77.897103	74.313863	35.315873
C	79.202341	74.911935	35.460610
C	79.005940	76.173376	35.934292
C	77.580483	76.347575	36.076679
C	75.609924	77.710270	36.691969
C	75.000800	78.945369	37.129290
C	73.655591	78.735137	37.143127
C	73.442310	77.373429	36.711500
C	71.975648	75.480219	36.115518
C	70.671099	74.878830	35.979425
C	70.868307	73.610725	35.523456
C	72.293529	73.436198	35.381557
C	72.902303	72.260246	34.956924
C	77.675262	73.009578	34.890150
C	76.978420	77.513765	36.535231
C	72.197763	76.773880	36.572742
C	75.938531	76.891270	33.143183
S	74.539724	75.831803	33.622503

H	76.035289	77.718418	33.855359
H	76.876817	76.331638	33.126803
H	75.729086	77.309748	32.154137
H	72.251911	71.427546	34.706694
H	78.545785	72.416509	34.626683
H	77.632578	78.342703	36.788931
H	71.325469	77.368150	36.827526
H	74.326901	69.939243	34.091136
H	77.004961	70.359330	34.047248
H	80.136624	74.414512	35.232937
H	79.745768	76.926600	36.174374
H	75.545481	79.845010	37.386800
H	72.867592	79.427778	37.410638
H	69.736674	75.376241	36.206518
H	70.129322	72.851289	35.301688
C	75.152733	76.438883	40.021649
C	74.619099	75.053230	39.741628
C	73.145229	74.822433	40.004172
C	72.629083	73.392291	39.739435
C	71.095372	73.357358	39.818071
C	73.239077	72.363874	40.704497
H	75.151105	76.637485	41.106234
H	74.543296	77.215506	39.547449
H	74.817690	74.771839	38.414155
H	75.267776	74.265984	40.138405
H	72.931668	75.085481	41.058101
H	72.563902	75.534472	39.400770
H	72.927122	73.120370	38.718178
H	70.711968	72.348869	39.620871
H	70.643722	74.035273	39.084410
H	70.744227	73.655782	40.815474
H	72.855070	71.358259	40.494745
H	72.986233	72.604950	41.746327
H	74.330436	72.319170	40.624921
H	76.184192	76.551439	39.670731

TSs of conformer 2:

ω

O	74.832237	73.231070	37.120084
Fe	74.646914	74.353493	35.802230
N	75.551608	73.117265	34.508312
N	76.418815	75.171660	36.264545
N	73.689451	75.723969	36.943627
N	72.828027	73.592506	35.265382
C	74.960823	72.109261	33.786762
C	75.960983	71.372088	33.052534
C	77.162980	71.940147	33.346545
C	76.898854	73.019858	34.266706
C	77.649390	74.807490	35.789358
C	78.682962	75.612698	36.401917
C	78.056200	76.470391	37.249966

C	76.640063	76.191296	37.151260
C	74.286094	76.671393	37.733624
C	73.285738	77.415077	38.460887
C	72.077066	76.898471	38.105893
C	72.338282	75.838312	37.162808
C	71.588827	73.996478	35.703577
C	70.561846	73.164041	35.124017
C	71.193256	72.253486	34.332846
C	72.608085	72.531513	34.426129
C	73.599194	71.833077	33.743665
C	77.880091	73.813667	34.847608
C	75.655411	76.891215	37.835642
C	71.355898	75.041055	36.587145
C	74.214240	75.157100	32.433073
S	74.145385	75.971868	34.057968
H	75.200699	74.725168	32.247016
H	73.468845	74.355032	32.391784
H	73.971910	75.896247	31.663908
H	73.281561	71.004976	33.116951
H	78.909852	73.625213	34.559482
H	75.981379	77.682822	38.503620
H	70.324530	75.249396	36.855033
H	75.753424	70.530244	32.404059
H	78.146663	71.659614	32.991879
H	79.741493	75.519356	36.194857
H	78.491459	77.231393	37.885239
H	73.498154	78.222307	39.150353
H	71.091697	77.193288	38.443871
H	69.500907	73.275836	35.308570
H	70.757580	71.464320	33.733038
C	74.331577	68.215875	40.279357
C	75.278919	69.147695	39.513234
C	74.575509	70.154881	38.587690
C	73.640883	71.176882	39.273857
C	72.909684	72.008669	38.241410
C	74.374986	72.073007	40.285797
H	73.675345	67.667411	39.591334
H	73.690296	68.767050	40.977140
H	75.923766	69.679444	40.225266
H	75.955253	68.538193	38.898498
H	75.330054	70.715301	38.020905
H	73.987241	69.594421	37.845279
H	72.865929	70.612646	39.825621
H	72.291122	72.818807	38.634998
H	72.414682	71.449729	37.443735
H	73.886035	72.718608	37.550903
H	73.681640	72.787374	40.746239
H	75.162855	72.645971	39.784256
H	74.830521	71.488271	41.092343
H	74.891828	67.476809	40.864170

ω-1

O	75.141104	74.718929	37.677615
Fe	74.772201	75.155242	36.040706

N	75.676479	73.518680	35.302652
N	76.503608	76.160540	35.960038
N	73.792681	76.844215	36.561569
N	72.980626	74.161828	36.023831
C	75.113998	72.280070	35.111478
C	76.118953	71.341297	34.673926
C	77.295513	72.024268	34.614186
C	77.011438	73.379289	35.020949
C	77.726147	75.671097	35.586168
C	78.737204	76.698051	35.713032
C	78.104967	77.813343	36.164172
C	76.707164	77.468573	36.309232
C	74.364869	78.061686	36.822242
C	73.356120	79.006504	37.237723
C	72.168153	78.341497	37.230306
C	72.450028	76.989392	36.812919
C	71.744328	74.673119	36.334799
C	70.736443	73.645753	36.211797
C	71.378422	72.510836	35.819477
C	72.779071	72.845690	35.700750
C	73.772634	71.961846	35.290988
C	77.968843	74.380267	35.137818
C	75.719022	78.357053	36.710813
C	71.493154	75.986963	36.708026
C	74.110066	74.444640	32.678035
S	74.057500	75.855888	33.825023
H	75.112552	74.014142	32.618781
H	73.413674	73.668825	33.015083
H	73.788377	74.792541	31.691744
H	73.473537	70.939024	35.081753
H	78.991048	74.125233	34.875116
H	76.027264	79.370238	36.950493
H	70.465277	76.254749	36.933417
H	75.932254	70.298541	34.450075
H	78.274803	71.657702	34.333555
H	79.785676	76.563073	35.479316
H	78.524344	78.788369	36.377768
H	73.549425	80.039417	37.498216
H	71.184337	78.714921	37.484818
H	69.680267	73.792363	36.399613
H	70.958418	71.532945	35.620301
C	76.630443	72.162502	39.043506
C	75.866984	72.265448	40.368793
C	75.081873	73.572284	40.575685
C	73.871052	73.842239	39.681699
C	73.111861	75.088940	40.104351
C	72.981730	72.650135	39.382915
H	77.333941	72.994787	38.929874
H	75.960584	72.196912	38.179676
H	75.189099	71.407446	40.473579
H	76.583170	72.177491	41.197617
H	74.715316	73.583093	41.618319
H	75.769178	74.425825	40.495101
H	74.416187	74.237073	38.504570

H	72.338869	75.357205	39.377387
H	73.785377	75.945448	40.218706
H	72.614822	74.923095	41.074461
H	72.133188	72.933164	38.752717
H	72.574050	72.235401	40.320437
H	73.522858	71.842883	38.879245
H	77.199829	71.225788	38.998347

ω-2R

O	75.332827	74.433051	37.583393
Fe	75.045480	74.974766	35.956772
N	75.257162	73.125483	35.202088
N	77.011447	75.298121	35.735751
N	74.769746	76.901835	36.502842
N	73.026430	74.681538	36.099246
C	74.278799	72.169556	35.075774
C	74.849204	70.937245	34.587844
C	76.184483	71.155912	34.433561
C	76.431449	72.520620	34.831623
C	77.951773	74.405985	35.295367
C	79.267075	75.007444	35.324719
C	79.104302	76.276980	35.781705
C	77.689362	76.451147	36.028914
C	75.752877	77.839863	36.681004
C	75.179113	79.080053	37.144691
C	73.837574	78.875362	37.255029
C	73.590484	77.510093	36.858983

C	72.081569	75.587636	36.515981
C	70.775171	74.972446	36.529432
C	70.941502	73.687638	36.110644
C	72.350850	73.517538	35.840671
C	72.930851	72.345083	35.366972
C	77.687923	73.113847	34.862652
C	77.111184	77.635638	36.464661
C	72.339223	76.905116	36.870407
C	73.912051	74.549308	32.672916
S	74.456921	75.886248	33.779651
H	74.690239	73.792451	32.547859
H	73.019482	74.068440	33.088443
H	73.650462	74.987934	31.705363
H	72.275353	71.493448	35.210962
H	78.531430	72.512224	34.537849
H	77.774518	78.475186	36.649515
H	71.495210	77.513214	37.181539
H	74.288025	70.030683	34.399466
H	76.946294	70.465730	34.093819
H	80.180201	74.507907	35.026787
H	79.855519	77.040939	35.937230
H	75.744017	79.978920	37.356876
H	73.073153	79.571237	37.576775
H	69.860407	75.474069	36.818876
H	70.191631	72.916601	35.985946
C	75.131809	75.792294	40.738523
C	73.734341	75.421365	40.223988

C	73.654126	74.109531	39.460605
C	74.191319	72.837434	40.103906
C	74.161656	71.656779	39.121630
C	73.406390	72.493084	41.389348
H	75.849062	75.816304	39.911472
H	75.497863	75.075604	41.482616
H	73.030080	75.383669	41.073185
H	73.365248	76.222924	39.572545
H	72.675604	73.957341	38.989775
H	74.454734	74.305972	38.371437
H	75.238250	73.009918	40.388473
H	74.565424	70.749808	39.588005
H	74.753158	71.879799	38.228915
H	73.133467	71.439241	38.802960
H	73.799694	71.575556	41.844260
H	72.344558	72.326835	41.166089
H	73.472004	73.291883	42.136211
H	75.118444	76.780726	41.212163

ω-2S

O	74.707261	73.373960	37.155599
Fe	74.612267	74.409512	35.764090
N	75.790477	73.203458	34.672331
N	76.223589	75.403275	36.422129
N	73.393738	75.742721	36.671674
N	72.958837	73.457592	35.021497
C	75.394948	72.126535	33.917836
C	76.548552	71.447931	33.378205
C	77.645015	72.119504	33.826576
C	77.161902	73.206453	34.643503
C	77.535685	75.117511	36.156815
C	78.401829	76.041261	36.856144
C	77.592173	76.892179	37.539976
C	76.231888	76.488560	37.257032
C	73.788260	76.787213	37.466741
C	72.634118	77.470777	37.998882
C	71.537559	76.818341	37.523889
C	72.020137	75.736353	36.700725
C	71.642342	73.762280	35.269594
C	70.778868	72.824859	34.590476
C	71.588322	71.955157	33.925774
C	72.947948	72.361224	34.199280
C	74.080681	71.734529	33.688658
C	77.980347	74.100764	35.322965
C	75.105256	77.138521	37.742199
C	71.200893	74.818564	36.054697
C	74.629480	75.033309	32.322265
S	74.234035	75.892623	33.876055
H	75.665788	74.687112	32.310226
H	73.969244	74.167072	32.202367
H	74.450172	75.722833	31.491952
H	73.926028	70.865250	33.056297
H	79.053226	73.984489	35.203102
H	75.264703	77.994284	38.391254

H	70.128300	74.941585	36.170147
H	76.507111	70.570466	32.745221
H	78.689908	71.906470	33.639611
H	79.483464	76.025746	36.812261
H	77.868525	77.723749	38.175790
H	72.675759	78.332716	38.652639
H	70.492557	77.033815	37.707091
H	69.696932	72.847324	34.624067
H	71.308336	71.115532	33.302081
C	72.638448	69.181124	37.903177
C	73.510246	70.383269	37.487604
C	72.994194	71.710169	38.013474
C	73.035161	71.989471	39.512380
C	72.478832	73.386782	39.830074
C	74.434514	71.807989	40.124994
H	71.603214	69.308349	37.563833
H	72.619392	69.052821	38.991414
H	74.540825	70.218524	37.824193
H	73.548806	70.429481	36.392662
H	73.829273	72.639379	37.461846
H	72.034755	71.993520	37.565497
H	72.362999	71.253242	39.991216
H	72.429341	73.551231	40.913190
H	71.468899	73.516487	39.422669
H	73.118810	74.161951	39.394784
H	74.415215	72.034009	41.197842
H	75.150869	72.482239	39.642858
H	74.804152	70.782834	40.011414
H	73.023998	68.252821	37.464769

ω-3R

O	74.845182	74.544806	37.767954
Fe	74.685571	75.046847	36.115146
N	75.197318	73.235504	35.429826
N	76.613908	75.597692	36.108445
N	74.124392	76.929744	36.617466
N	72.709320	74.534122	36.018097
C	74.357540	72.167491	35.230216
C	75.117289	71.005449	34.834651
C	76.425097	71.382200	34.804399
C	76.466621	72.772413	35.190153
C	77.694782	74.827920	35.775796
C	78.918723	75.578230	35.950926
C	78.558361	76.812001	36.393507
C	77.114383	76.815999	36.482827
C	74.968716	77.966203	36.917247
C	74.209231	79.131490	37.303311
C	72.894674	78.782985	37.236611
C	72.850784	77.404240	36.812855
C	71.621085	75.332517	36.282413
C	70.402081	74.571791	36.142280
C	70.765568	73.306673	35.793559
C	72.208587	73.294898	35.716032
C	72.974036	72.187648	35.361901

C	77.633047	73.513242	35.332467
C	76.357524	77.917258	36.860474
C	71.681699	76.669020	36.652179
C	73.961179	74.497774	32.744246
S	74.259101	75.895055	33.870493
H	74.820031	73.822551	32.714400
H	73.085187	73.932673	33.081704
H	73.754729	74.894011	31.745708
H	72.443894	71.261940	35.158309
H	78.571214	73.016270	35.104864
H	76.895811	78.822564	37.124771
H	70.741848	77.179695	36.838895
H	74.689535	70.035527	34.614200
H	77.293822	70.785088	34.557622
H	79.911572	75.193405	35.755375
H	79.193079	77.654908	36.636030
H	74.641238	80.083508	37.584735
H	72.024128	79.389202	37.452899
H	69.406520	74.969353	36.293695
H	70.129867	72.452238	35.598092
C	73.720473	75.231749	40.635739
C	73.016396	74.503617	39.512117
C	72.743266	73.023839	39.691823
C	71.684000	72.666463	40.769371
C	71.598188	71.141990	40.935093
C	70.304393	73.257513	40.444152
H	74.635881	74.707153	40.934437
H	73.079813	75.311420	41.528385
H	72.182011	75.065413	39.083443
H	73.907263	74.552757	38.493356
H	72.414925	72.596032	38.734199
H	73.685139	72.520328	39.951712
H	72.019149	73.089530	41.727852
H	70.883211	70.867207	41.720337
H	72.571767	70.713108	41.200831
H	71.267901	70.665130	40.002732
H	69.562973	72.961474	41.196276
H	69.949792	72.904581	39.466577
H	70.325905	74.352418	40.412076
H	73.994687	76.249632	40.338218

ω-3S

O	75.407122	73.314344	37.084631
Fe	74.987122	74.334519	35.747733
N	76.258872	73.389720	34.508624
N	76.438212	75.610519	36.279401
N	73.638730	75.395486	36.811592
N	73.482596	73.087320	35.140883
C	76.004079	72.262476	33.767289
C	77.206085	71.825057	33.099281
C	78.192481	72.692907	33.457592
C	77.594168	73.657762	34.348234
C	77.747076	75.586261	35.878386
C	78.487751	76.652276	36.517146

C	77.604513	77.324121	37.301536
C	76.324583	76.668806	37.140626
C	73.904377	76.486213	37.597489
C	72.703381	76.920028	38.269538
C	71.711698	76.067628	37.890802
C	72.304974	75.114132	36.984840
C	72.167831	73.120562	35.536483
C	71.438245	72.032715	34.928565
C	72.326937	71.344657	34.159665
C	73.600611	72.012556	34.298151
C	74.774024	71.622625	33.660461
C	78.290713	74.685490	34.973304
C	75.149900	77.084280	37.752447
C	71.616803	74.058623	36.399193
C	74.556275	74.990205	32.337214
S	74.142014	75.731299	33.946022
H	75.635288	74.861203	32.223194
H	74.075102	74.009666	32.249544
H	74.166161	75.639180	31.547554
H	74.726654	70.742513	33.025932
H	79.348298	74.780500	34.746407
H	75.207965	77.946839	38.409417
H	70.560742	73.962981	36.632725
H	77.270573	70.961612	32.449165
H	79.234649	72.688282	33.164309
H	79.542350	76.845884	36.367292
H	77.780117	78.186752	37.931747
H	72.645851	77.764906	38.944046
H	70.670975	76.068215	38.189170
H	70.383721	71.837932	35.077683
H	72.152495	70.468349	33.548072
C	75.269154	70.243577	37.832112
C	74.164546	71.274077	37.911601
C	73.729139	71.739527	39.286997
C	73.012205	70.671913	40.156906
C	71.698553	70.192428	39.522622
C	72.764955	71.223567	41.568775
H	74.933530	69.262228	38.203416
H	76.134935	70.544745	38.433799
H	74.729260	72.383313	37.373465
H	73.338914	71.082331	37.221072
H	74.613221	72.095409	39.834283
H	73.059047	72.604787	39.181677
H	73.682868	69.804862	40.248337
H	71.205461	69.445982	40.157169
H	71.859683	69.738658	38.538565
H	71.001088	71.030822	39.392322
H	72.289418	70.472318	42.211319
H	72.104339	72.100034	41.536249
H	73.702152	71.531688	42.047333
H	75.606118	70.103730	36.799098

The coordinates of the ONIOM QM region and the single point energies (a.u.) of each species

RC, -2779.595686028

(ZPE = 14.221967)

C	50.7044830	37.5963110	33.9863690
H	49.9574220	38.3917700	33.8562400
H	50.5288340	36.8729340	33.1773020
C	50.4618940	36.9087100	35.3453990
H	51.2136000	36.1196730	35.4951790
C	50.5607740	37.8962080	36.5194020
H	49.8361340	38.7118390	36.4018490
H	50.3503930	37.3958270	37.4728950
H	51.5569960	38.3441910	36.5979790
C	49.0789900	36.2394340	35.3269610
H	48.2952450	36.9898600	35.1539200
H	49.0065490	35.4853530	34.5334900
H	48.8543890	35.7487760	36.2821040
C	42.7175330	36.7789540	30.3029100
H	42.3990590	36.0359030	29.5621880
H	43.4611980	37.4164310	29.8109650
C	43.3263840	36.1119390	31.5170700
C	44.5920390	36.4910130	31.9812110
H	45.1610840	37.2325010	31.4234780
C	45.1256930	35.9418940	33.1504930
H	46.1191040	36.2310960	33.4826510
C	44.3907390	35.0070830	33.8802710
H	44.7987300	34.5764360	34.7909320
C	43.1305600	34.6117980	33.4226820
H	42.5554970	33.8740040	33.9780410
C	42.6083370	35.1537600	32.2494380
H	41.6254090	34.8381350	31.9027840
C	44.2350590	41.3306430	32.3115170
H	44.0001310	41.8620490	31.3838430
H	44.3956200	42.0797930	33.0904460
C	45.5024340	40.4616270	32.1425160
H	45.5399940	39.6417790	32.8692070
H	45.5040230	39.9758290	31.1559950
C	46.8255820	41.1923780	32.2802170
O	47.8806570	40.6746430	32.5809530
O	46.7088940	42.5275190	32.0328060
C	41.3549600	41.8105320	39.6575650
H	41.5447760	42.5561210	40.4481370
C	42.2606190	40.6018440	39.9130950
H	42.0898270	39.7997250	39.1850870
H	43.3157030	40.8962860	39.8368160
H	42.1130520	40.1898330	40.9199240
O	41.5836150	42.3817740	38.3724420
H	42.5445900	42.3423370	38.2045770
S	46.2729370	46.9045210	37.1507580
C	48.8959490	43.5932390	39.7557780
C	47.9700940	43.8054430	40.7219510
C	46.7422690	44.1751020	40.0633210
C	45.5410700	44.4677120	40.6885630
H	45.5077640	44.4124970	41.7705060
C	48.2429750	43.8036260	38.4878830
C	48.8462740	43.6262900	37.2502560

H	49.8738770	43.2790920	37.2563110	H	44.3499870	40.4241580	36.1303250
N	46.9322370	44.1715970	38.6952450	H	43.5295230	38.9035820	36.5443120
Fe	45.4900480	44.4646760	37.2835420	C	45.5754580	39.0331630	37.2888460
O	45.0039640	42.9158960	37.3018260	C	46.9045890	39.6941270	36.8763930
N	44.1916300	45.0572740	35.8643060	H	47.6836530	39.5478000	37.6329990
N	44.1727900	44.9732610	38.7109270	H	46.7637180	40.7687060	36.7298540
C	44.3557340	44.8275130	40.0617450	H	47.2701990	39.2588850	35.9350430
C	43.1258210	45.1252600	40.7593100	H	45.2622020	39.4925670	38.2399090
C	42.2203520	45.4981630	39.8160640	C	45.6985480	37.5159200	37.5139250
C	42.8779800	45.3855590	38.5334780	H	46.0094240	37.0351430	36.5730210
C	42.2875540	45.6406590	37.2991090	H	44.7000990	37.1106300	37.7396700
H	41.2478640	45.9573470	37.3101140	C	46.6610370	37.0988820	38.6362740
C	42.8887800	45.4723610	36.0601610	H	46.3452470	37.5905460	39.5678790
C	42.2586470	45.6845480	34.7791040	H	47.6601910	37.4898750	38.4093670
C	43.1719000	45.3782260	33.8246340	C	46.7203750	35.5746940	38.8213580
C	44.3891720	45.0073400	34.5033620	H	47.0291450	35.0956100	37.8819550
C	45.5554390	44.6013020	33.8659410	H	45.7161980	35.1852400	39.0363060
H	45.5155040	44.5289840	32.7847060	H	51.7141534	38.0451210	33.8224356
C	46.7251900	44.1993120	34.4861370	H	41.8287052	37.4057676	30.5415832
C	47.8953000	43.6896990	33.7790210	H	43.3509626	40.7284784	32.6129808
C	47.8743730	43.3396110	32.3157470	H	40.2797511	41.5172532	39.7386366
H	47.7384310	44.2178750	31.6790510	H	47.3667590	47.2571127	37.9560169
H	48.7721150	42.8039210	32.0090720	H	49.9187992	43.2464484	39.8927828
N	46.9300700	44.2083440	35.8427280	H	48.1240363	43.6959154	41.7886745
C	48.2311300	43.7875740	36.0222370	H	42.9943235	45.0494018	41.8380724
C	48.8267730	43.4692070	34.7479870	H	41.2187486	45.6100468	39.9291632
C	44.4901490	39.3452330	36.2480600	H	41.2441172	46.0305409	34.6184158
H	44.7512560	38.9155630	35.2698290	H	43.0901653	45.5824169	32.8339750

H	49.8365534	43.0638663	34.6235655
H	47.4096549	35.2308532	39.6341495

TS$_{\omega}$, -2779.564115106

(ZPE = 14.215015)

C	50.7831320	37.5105790	34.0493540
H	49.9996900	38.2767240	33.9709620
H	50.6040140	36.7995220	33.2304630
C	50.6450000	36.7797740	35.4034300
H	51.5093120	36.1163840	35.5516340
C	50.5986490	37.7654090	36.5820540
H	49.7209770	38.4204220	36.5051930
H	50.5337510	37.2327010	37.5380160
H	51.4874280	38.4041480	36.6225150
C	49.3942190	35.8889090	35.3914880
H	48.4861140	36.4858990	35.2309870
H	49.4452280	35.1398710	34.5917260
H	49.2764980	35.3539470	36.3421150
C	42.6074190	36.7694020	30.3555950
H	42.2563430	36.0536290	29.6033560
H	43.3872200	37.3736980	29.8776240
C	43.1692810	36.0536300	31.5646000
C	44.4494870	36.3595440	32.0422240
H	45.0650110	37.0658000	31.4893070
C	44.9418670	35.7745140	33.2109870
H	45.9419800	36.0172650	33.5587690
C	44.1552830	34.8684050	33.9221560
H	44.5338540	34.4050450	34.8295280
C	42.8818140	34.5411260	33.4485920
H	42.2657850	33.8269830	33.9907070
C	42.3965670	35.1252850	32.2795790
H	41.4005500	34.8659490	31.9234580
C	44.2462050	41.3834220	32.2598520
H	43.9820180	41.9019480	31.3325300
H	44.4197000	42.1446440	33.0244660
C	45.5176560	40.5219090	32.0740820
H	45.5517550	39.6861750	32.7801700
H	45.5279530	40.0559360	31.0787890
C	46.8453740	41.2380770	32.2414960
O	47.8816960	40.7078920	32.5869860
O	46.7580980	42.5698140	31.9831300
C	41.3469700	41.8114530	39.6997670
H	41.5087280	42.5714120	40.4830940
C	42.2550540	40.6151940	40.0032590
H	42.1119800	39.7984870	39.2851050
H	43.3074430	40.9213670	39.9509740
H	42.0837200	40.2184970	41.0126380
O	41.6081780	42.3605310	38.4120070
H	42.5776190	42.3515680	38.2801580
S	46.2386070	46.8725110	37.0591120
C	48.9189450	43.5576900	39.8040410
C	47.9865380	43.7461110	40.7694210
C	46.7550370	44.1058110	40.1108840
C	45.5514220	44.4117190	40.7355490
H	45.5151500	44.3437550	41.8163750
C	48.2658740	43.7584160	38.5353750

C	48.8703490	43.5941630	37.2933790	H	45.3781120	39.7536880	35.3786860
H	49.9087410	43.2795250	37.2969600	H	44.1579260	40.7843990	36.1563460
N	46.9481320	44.1022550	38.7452600	H	43.9201240	39.0235420	36.0653140
Fe	45.5101550	44.4612190	37.3457160	C	45.3893610	39.6396070	37.5565720
O	44.9336410	42.8147910	37.4402570	C	46.4036890	40.7532690	37.7643150
N	44.1808500	44.9888800	35.9201430	H	46.8186380	40.8447140	38.7702180
N	44.1915520	44.9651730	38.7655750	H	45.6880130	41.9484810	37.6018850
C	44.3729440	44.8008150	40.1167610	H	47.1642290	40.8329600	36.9827140
C	43.1380870	45.0931130	40.8134390	H	44.6428890	39.7106650	38.3604420
C	42.2355600	45.4736030	39.8722940	C	46.0078430	38.2199730	37.6227940
C	42.8981070	45.3741360	38.5894980	H	46.7488710	38.1129200	36.8168940
C	42.3088890	45.6445080	37.3568670	H	45.1990470	37.5135520	37.3792310
H	41.2749600	45.9775260	37.3813830	C	46.6460330	37.7681820	38.9511320
C	42.9014970	45.4739510	36.1149530	H	45.9912500	38.0552590	39.7855940
C	42.2875980	45.7331650	34.8299960	H	47.5934260	38.3006030	39.1053810
C	43.1912650	45.3987750	33.8799950	C	46.8575010	36.2399340	38.9290300
C	44.3850870	44.9467920	34.5569000	H	47.3391730	35.9589370	37.9831610
C	45.5437150	44.5177330	33.9257530	H	45.8779750	35.7435140	38.9215870
H	45.5109980	44.4569760	32.8429700	H	51.7707601	38.0007722	33.8584045
C	46.7208110	44.1191030	34.5433830	H	41.7502931	37.4389090	30.5967967
C	47.8992040	43.6496610	33.8224040	H	43.3740223	40.7783822	32.5955730
C	47.9075550	43.3710310	32.3427880	H	40.2728347	41.5058935	39.7595332
H	47.7910840	44.2786450	31.7449000	H	47.3352940	47.1549624	37.9037109
H	48.8148460	42.8514190	32.0372750	H	49.9458830	43.2240430	39.9412521
N	46.9341380	44.1219230	35.8950530	H	48.1514479	43.6584701	41.8366063
C	48.2468120	43.7313150	36.0654050	H	43.0008252	45.0142181	41.8913154
C	48.8435830	43.4395210	34.7836810	H	41.2371996	45.5700825	39.9778127
C	44.6638760	39.8174770	36.2102680	H	41.3392878	46.2311559	34.6578422

H	43.1191869	45.6155738	32.8966900
H	49.8625995	43.0572527	34.6507553
H	47.4627225	35.7980906	39.7647383

$TS_{\omega-1}$, -2779.562333535

(ZPE = 14.215046)

C	50.7338450	37.4029420	34.0258080
H	49.9463010	38.1688760	34.0097600
H	50.5581240	36.7601290	33.1514000
C	50.5939650	36.5542930	35.3095560
H	51.4327940	35.8463170	35.3694240
C	50.6138740	37.4135590	36.5826960
H	49.7733880	38.1190390	36.5890970
H	50.5276970	36.7873260	37.4793450
H	51.5362330	37.9964320	36.6739820
C	49.3070870	35.7180390	35.2449590
H	48.4204000	36.3632170	35.1777250
H	49.3038490	35.0558800	34.3705930
H	49.1945790	35.0921480	36.1388690
C	42.5923580	36.7062660	30.3651870
H	42.2412810	36.0017350	29.6033230
H	43.3929660	37.2983150	29.9067280
C	43.1088620	35.9782000	31.5881620
C	44.3696030	36.2784460	32.1185280
H	45.0140590	36.9760440	31.5881900
C	44.8022530	35.7054530	33.3166730
H	45.7852940	35.9482950	33.7100870
C	43.9751250	34.8178730	34.0054910
H	44.3039490	34.3764100	34.9419350
C	42.7244490	34.4899840	33.4755690
H	42.0752710	33.7926020	33.9993130
C	42.2993420	35.0611510	32.2768950
H	41.3171850	34.8068500	31.8811920
C	44.2429550	41.3389830	32.2653460
H	43.9462690	41.8622890	31.3507210
H	44.4118770	42.0978240	33.0337080
C	45.5333380	40.5114270	32.0385030
H	45.5821330	39.6375840	32.6947300
H	45.5542420	40.1069620	31.0173120
C	46.8470780	41.2423360	32.2496440
O	47.8723880	40.7260100	32.6474490
O	46.7647930	42.5665520	31.9594930
C	41.3315740	41.7151100	39.7642200
H	41.4994580	42.4722270	40.5487790
C	42.2074160	40.4971220	40.0800210
H	42.0618030	39.6872360	39.3544310
H	43.2681150	40.7769470	40.0540470
H	42.0057380	40.0965150	41.0825890
O	41.6195220	42.2568780	38.4782780
H	42.5919670	42.2641320	38.3763980
S	46.2292100	46.8771820	37.0598940
C	48.9748550	43.6305400	39.7618350
C	48.0391830	43.7727700	40.7321700
C	46.7943910	44.1035730	40.0839970
C	45.5841520	44.3603980	40.7188370
H	45.5555740	44.2885890	41.7999750

C	48.3112940	43.8280200	38.4973270	C	45.1580300	40.0647720	35.9109310
C	48.9177300	43.7026860	37.2533400	H	45.3100120	39.0043900	35.6430630
H	49.9672840	43.4282280	37.2534230	H	45.3122170	40.6583210	35.0060660
N	46.9828500	44.1261240	38.7187570	H	44.1125070	40.1729000	36.2197740
Fe	45.5272790	44.4295230	37.3277630	C	46.1084840	40.4840220	37.0156280
O	45.0220680	42.7718940	37.3876460	C	47.5612480	40.5706210	36.5860170
N	44.1955810	44.9720060	35.9133230	H	48.1925640	41.0044170	37.3663830
N	44.2054880	44.8814890	38.7535210	H	47.6789290	41.1398230	35.6620660
C	44.3951460	44.7198730	40.1053410	H	47.9440350	39.5571570	36.3819030
C	43.1615840	45.0067870	40.8078740	H	45.6961510	41.7724330	37.2208370
C	42.2564000	45.3934840	39.8733780	C	45.8712360	39.7873150	38.3516080
C	42.9118140	45.3018570	38.5859180	H	44.7922860	39.6288030	38.4763060
C	42.3232010	45.6021690	37.3627580	H	46.1851400	40.4378940	39.1776950
H	41.2921860	45.9434890	37.3950830	C	46.6023230	38.4176120	38.5135500
C	42.9162960	45.4526900	36.1175860	H	47.6156650	38.6084850	38.8876910
C	42.2969480	45.7190340	34.8375360	H	46.7207480	37.9425530	37.5315030
C	43.1937610	45.3838630	33.8805900	C	45.8198030	37.4791920	39.4508270
C	44.3912790	44.9303060	34.5467580	H	44.9555960	37.0805030	38.9039200
C	45.5434240	44.5029090	33.9052940	H	45.3990260	38.0758930	40.2677180
H	45.5009970	44.4370350	32.8231950	H	51.7174461	37.9159122	33.8724043
C	46.7308950	44.1237290	34.5155260	H	41.7441279	37.3901537	30.5974762
C	47.9133260	43.6736560	33.7896180	H	43.3914971	40.7086455	32.6117537
C	47.9136260	43.3738930	32.3140090	H	40.2500000	41.4351384	39.8062025
H	47.7938680	44.2740970	31.7055290	H	47.3249862	47.1746799	37.9021396
H	48.8210590	42.8525470	32.0116860	H	50.0030764	43.2964065	39.8904884
N	46.9571530	44.1477580	35.8635320	H	48.2104732	43.6784144	41.7978648
C	48.2819650	43.8128050	36.0273050	H	43.0277230	44.9273289	41.8859292
C	48.8755290	43.5173540	34.7445200	H	41.2599441	45.5000183	39.9864794

H	41.3485191	46.2189106	34.6709785	H	49.8905496	43.1261342	34.6161830
H	43.1144376	45.5994080	32.8975818	H	46.3826483	36.6086959	39.8958113
